# Signaling pathways regulating cardiac regeneration

**DOI:** 10.1186/s13619-026-00297-7

**Published:** 2026-07-28

**Authors:** Jianan Li, Minjie Hu, Lu Ding, Yongyu Wang

**Affiliations:** Department of Cell Biology, Institute of Hypoxia Medicine, School of Basic Medical Sciences, Wenzhou Medical University, Wenzhou, Zhejiang 325035 China

**Keywords:** Cardiac regeneration, Signaling pathways, Transcriptional regulation, Cell cycle proteins, Metabolic reprogramming, Therapeutic delivery, Cardiomyocyte proliferation

## Abstract

Cardiac regeneration represents a pivotal frontier in addressing cardiovascular diseases, the leading global cause of mortality. This review integrates current advancements in understanding the molecular mechanisms driving cardiomyocyte proliferation and myocardial repair. Key signaling pathways—including Hippo/YAP, Wnt/β-catenin, NRG1-ErbB, MAPK, and Notch—orchestrate cardiomyocyte dedifferentiation, cell cycle re-entry, and tissue remodeling. Hippo inhibition promotes cardiomyocyte proliferation and cytoskeletal reorganization, while Wnt/β-catenin exhibits dual roles depending on developmental context and injury phase. NRG1-ErbB and MAPK/ERK pathways integrate metabolic reprogramming and paracrine signaling to enhance regeneration. Transcriptional regulators such as Meis1, GATA4, and Tbx20 modulate cell cycle dynamics, while extracellular matrix components (e.g., Agrin, FSTL1, POSTN) and growth factors (PDGF, FGF, VEGF, Ang-1) reshape the regenerative microenvironment. Despite progress, challenges persist in spatiotemporal control of proliferation, interspecies pathophysiological disparities, and therapeutic delivery precision. Emerging technologies—engineered myocardial grafts, transient modified mRNA systems (e.g., SMRTs), and hypoxia-mediated metabolic switching—highlight translational potential. Future strategies demand integration of multi-omics, biomaterials, and combinatorial interventions to bridge mechanistic insights with clinical applications.

## Background

Cardiovascular disease remains the leading global cause of mortality, with ischemic heart disease-induced myocardial infarction (MI) and subsequent heart failure (HF) posing significant challenges in clinical management (Roth et al. 2020). Following MI, ischemia and hypoxia trigger extensive necrosis of functional cardiomyocytes (CMs) in mammalian hearts, culminating in fibrotic scar formation. This irreversible damage compromises cardiac contractility and initiates pathological remodeling, ultimately progressing to HF (Prabhu and Frangogiannis 2016). While current therapies (e.g., revascularization, beta-blockers, ACE inhibitors) mitigate disease progression, they fail to regenerate lost CMs or reverse fibrosis (McMurray and Pfeffer, 2005). Consequently, elucidating the molecular mechanisms of cardiac regeneration, whether through endogenous repair pathways (Porrello et al. 2011) or exogenous interventions (Sadek and Olson 2020), has emerged as a pivotal frontier in cardiovascular research.

**Fig. 1:**
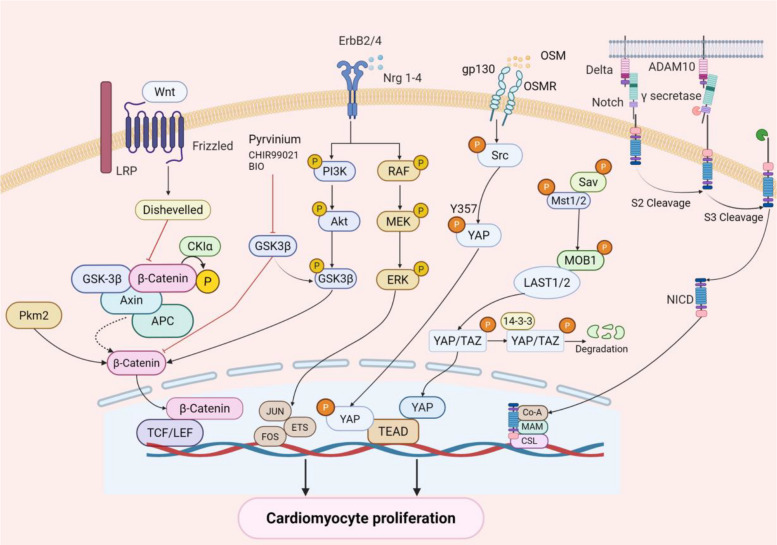
Overview of signaling pathways regulating CM proliferation. (Right) Notch signaling activation: Upon binding of Delta ligand, the Notch receptor is activated by sequential protein hydrolysis (S2 by ADAM10 metalloproteinase and S3 by γ-secretase), releasing the Notch intracellular structural domain (NICD).The NICD translocates to the nucleus, replaces co-inhibitory factors with CSL proteins (Su (H) or Lag-1), and recruits Co-activator (Co-A) and Mastermind-like protein (MAM) to form a transcriptional complex and regulate the expression of target genes, thereby regulating proliferation. (Left) Wnt/β-catenin signaling cascade: Wnt ligands bind Frizzled-LRP receptor complexes, triggering Dishevelled-mediated inhibition of the β-catenin destruction complex (GSK3β/APC/Axin). Stabilized β-catenin accumulates in the cytoplasm, translocates to the nucleus, and associates with TCF/LEF transcription factors to activate pro-proliferative genes. (Center) ErbB2/4 binds to Nrg 1–4 and activates downstream signaling through the PI3K/Akt and RAF/MEK/ERK pathways. Glycoprotein 130 (gp130) binds to oncostatin M (OSM) and OSMR, activates Src and YAP, and promotes CM proliferation. Symbols: Arrows (→) denote activation or positive regulation. Blunt ends (┴) denote inhibition or negative regulation. Dashed lines represent indirect regulation or translocation. Small molecule inhibitors/activators are indicated (e.g., CHIR99021, Pyrvinium). Created in https://BioRender.com

Divergent cardiac regenerative capacities across species offer critical insights. Fish, amphibians and neonatal mammals (such as mice and pigs) (Weinberger and Riley 2024; Ye et al. 2018) achieve robust cardiac regeneration post-injury via CM dedifferentiation, proliferation, and redifferentiation, whereas adult mammalian CMs lose regenerative potential due to cell cycle exit and suppression of pro-regenerative signaling (Bishop et al. 2022; Porrello et al. 2011; Poss et al. 2002). Central to this process are evolutionarily conserved pathways such as Hippo-YAP, Wnt/β-catenin, NRG1/ErbB, Mitogen-activated protein kinases (MAPK), and Notch, which orchestrate CM proliferation, apoptosis, metabolic adaptation, and extracellular matrix remodeling to determine repair outcomes (Fig.1).

Despite progress, translating cardiac regeneration from bench to bedside faces multifaceted challenges. Key questions persist: How can crosstalk between signaling pathways be harnessed to enhance regenerative efficiency? Given the limited regenerative capacity of human CMs, how do fundamental mechanistic divergences between regenerative models (e.g., zebrafish) and human pluripotent stem cell-derived CMs impede the identification of clinically viable therapeutic targets for heart regeneration? Advances in single-cell sequencing, CRISPR-Cas9 gene editing, and artificial intelligence (AI)-driven drug discovery are now unraveling the complexity of cardiac regeneration networks, accelerating preclinical validation of novel targets.

This review integrates current knowledge on core signaling pathways governing cardiac regeneration, highlighting breakthroughs in mechanistic understanding, regulatory interplay, and therapeutic modulation. We further evaluate translational prospects and hurdles, aiming to bridge mechanistic insights with innovative strategies for clinical intervention.

## The key signaling pathways for cardiac regeneration

### Hippo/YAP signaling

The Hippo signaling pathway is an evolutionarily conserved regulator of organ size and tissue homeostasis, consisting of three core modules: a central kinase cascade formed by mammalian Ste20-like kinases 1 and 2 (MST1/2) and large tumor suppressor kinases 1 and 2 (LATS1/2) in complex with the adaptor proteins SAV1 and MOB1; upstream regulators such as FAT, FRMD , and NF2; and downstream effectors including Yes-associated protein (YAP)/transcriptional co-activator with PDZ-binding motif (TAZ) in association with TEAD family members 1–4 (TEAD 1–4) and WBP2.Upon activation by upstream signals or RASSF1A binding, MST1/2 kinases phosphorylate and activate the LATS1/2–SAV1 complex. Together with MOB1, LATS1/2 then phosphorylates YAP/TAZ, leading to its cytoplasmic retention via 14–3-3 binding or promoting its degradation. When the Hippo pathway is inactivated, unphosphorylated YAP/TAZ accumulates in the nucleus, where it forms heterodimers with TEAD and other transcriptional cofactors to activate genes that promote cell proliferation and survival (Huang et al. 2005; Moya and Halder 2019; Xie et al. 2022).

The Hippo pathway plays critical roles in heart development, as well as in adult mammalian heart regeneration after injury. When knockout of embryonic CM-specific *Sav1* to inactivate Hippo signaling, it causes CM hyperproliferation, cardiac hypertrophy, and postnatal lethality (Heallen et al. 2011). Conversely, in adult mammalian hearts, such as those of mice, Hippo pathway deficiency promotes CM regeneration after MI. Heallen et al. showed that CM-specific mutants of *Sav1* and *Lat1/2* induce cell cycle re-entry and cytokinesis, thereby improving functional recovery following MI or apical resection (Heallen et al. 2013). Notably, in *Sav1* mutant mice with cardiac-specific Hippo deficiency, YAP activation upregulates genes involved in cytoskeletal remodeling—including *ACTRT2*, *PKP4*, *ENAH*, and *FMN2*—suggesting that enhanced cytoskeletal reorganization may contribute to heart regeneration, in addition to CM proliferation (Morikawa et al. 2015).

As a core downstream effector of the Hippo pathway, YAP has been established as a key regulator of cardiac regeneration in mice (Xin et al. 2013). YAP stimulates CM proliferation by activating genes involved in DNA replication and mitosis, as well as by promoting cell cycle-related transcription factors. It also enhances cell survival through upregulation of connective tissue growth Factor (*CTGF*), cysteine-rich angiogenic inducer 61 (CYR61), AXL Receptor Tyrosine Kinase , and anti-apoptotic genes (Johnson and Halder 2014; Kapoor et al. 2019; Totaro et al. 2018; Zanconato et al. 2015). Following injury, activated YAP further supports CM survival and regeneration by engaging transcriptional complexes such as TEAD–PITX2 or forkhead box O1 (FOXO1) to initiate protective gene programs (Shao et al. 2014). YAP deficiency disrupts CM proliferation, leading to cardiac insufficiency and embryonic lethality. Xin et al*.* demonstrated that fetal inactivation of *Yap1* causes lethal myocardial hypoplasia, whereas *Yap1* activation enhances trabecular CM proliferation via YAP1–TEAD interactions without altering physiological hypertrophy (Xin et al. 2011). Thus, YAP serves as a central mediator of the Hippo pathway and is a critical regulator of both cardiac development and postnatal CM proliferation.

Therapeutic strategies targeting YAP, a key effector of the Hippo pathway, have shown considerable promise in enhancing cardiac regeneration and repair. For instance, adeno-associated virus serotype 9 (AAV9)-mediated delivery of phosphorylation-resistant YAP S127A has been shown to reduce scarring and promote neovascularization after MI (Xin et al. 2013). Similarly, sustained expression of human YAP reactivates proliferation in adult CMs, leading to improved cardiac repair (Lin et al. 2014). The constitutively active mutant YAP5SA not only induces proliferation in adult CMs but also triggers Hippo-mediated negative feedback to prevent excessive cell division (Monroe et al. 2019). Transient expression of YAP5SA via a CM-specific modified mRNA translation system (CM-SMRTs) after MI enhances regeneration, reduces infarct size, and limits off-target effects (Wang et al. 2024a). Moreover, the novel Drug-Elicitable Alternative-splicing Module (DreAM) enables controllable, AAV-mediated YAP5SA delivery, supporting safe and transient YAP activation in CMs to stimulate regeneration with minimal off-target risks (Chen et al. 2025).

YAP activity is also modulated by post-translational modifications. After MI, YAP acetylation at lysine 265 (K265) by CREB-binding protein/E1A-binding protein p300 (CBP/P300) increases its binding to tubulin alpha 4a (TUBA4A) , leading to microtubule-dependent cytoplasmic retention and suppression of regenerative responses. The K265-to-arginine (K265R) mutation prevents this sequestration, maintaining YAP nuclear localization and restoring regenerative capacity (Liu et al. 2025). Furthermore, YAP is inactivated through Hippo-mediated phosphorylation and reactivated via dephosphorylation by protein phosphatase 1 and 2 A (PP1/PP2A), which facilitates its nuclear translocation and transcriptional activity (Haemmerle et al. 2017). Beyond its core Hippo pathway functions, YAP also integrates signals from other pathways to support heart regeneration. Toll-like receptor 3 (TLR3) promotes CM proliferation through glycolysis-mediated upregulation of miR-152, a process dependent on YAP1 signaling that inhibits the DNA Methyltransferase 1 (DNMT1)–p27Kip1 axis. Inhibition of glycolysis (e.g., using 2-DG) suppresses YAP1 activity and impairs cardiac repair (Wang et al. 2018b). In addition, macrophage-derived OSM enhances regeneration by activating the CM gp130–Src–YAP axis, with YAP phosphorylation at Y357 playing a critical role. Adenovirus-mediated constitutive activation of gp130 has also been shown to drive cardiac regeneration in adult mice post-MI (Li et al. 2020b).

These findings highlight the therapeutic potential of targeting YAP regulatory networks. For example, cannabidiol promotes cardiac repair and reduces infarct size in MI mice by activating the miR-143-3p/Yap/catenin delta 1 axis (Ren et al. 2024). Conversely, β-adrenergic receptor signaling inhibits heart regeneration by downregulating m6A modification of *Yap* (Guan and Li 2025).

In summary, inhibition of the Hippo pathway promotes cardiac regeneration through multiple mechanisms involving YAP activation, epigenetic regulation, and metabolic reprogramming. Following cardiac injury, modulation of Hippo signaling not only stimulates CM proliferation but also influences extracellular matrix remodeling and immune responses, collectively supporting myocardial regeneration. These insights establish a strong foundation for developing Hippo pathway-targeted therapies to improve cardiac repair.

### Dual roles of Wnt/β-catenin signaling in cardiac regeneration

The Wnt signaling network comprises canonical (β-catenin-dependent) and non-canonical (β-catenin-independent) pathways. The canonical pathway drives β-catenin nuclear translocation to activate the T cytokine/lymphoid enhancer binding factor (TCF/LEF) transcription factors, primarily regulating cell proliferation, while non-canonical pathways govern cell polarity and migration. These pathways form an interconnected regulatory network which is critical for cardiac development and repair (Liu et al. 2022; Niehrs 2012). The canonical Wnt signaling pathway is primarily orchestrated by Wnt ligands, Frizzled receptors, Low-density lipoprotein receptor-related protein 5/6 (LRP5/6) co-receptors, the Axin/APC/GSK3β/CK1 destruction complex, and nuclear β-catenin (Li et al. 2021). This pathway plays a critical role in regulating CM proliferation, with context-dependent effects observed across developmental stages and injury models.

In neonatal mice (in vivo) and human stem cell-derived CMs (in vitro), Wnt/β-catenin signaling promotes CM proliferation, whereas in adult mice it provides only cardioprotection, preventing reactivation of pro-proliferation gene program (Quaife-Ryan et al. 2020). Thus, in adult heart, inhibition of canonical Wnt pathways by small molecules, such as Cardiomogen 1 and 2, Pyrvinium, or ICG001 could promote cardiac regeneration (Saraswati et al. 2010; Sasaki et al. 2013; Xie et al. 2020). In adult zebrafish, the heart could regenerate after cardiac apex resection, which is mediated by the expression and secretion of Wnt inhibitors Dkk3/sFrp1 from the epicardium and fibroblasts, or *Dkk1/sFrp2* in the myocardium (Peng et al. 2020). Consistently, overexpression of *dkk1* to block Wnt signaling promotes zebrafish CM proliferation, while ectopic activation of *wnt8* signaling impedes CM proliferation after injury (Peng et al. 2021). Conversely, non-canonical *wnt2bb* activates JNK1/CREB1 to stimulate zebrafish CM proliferation, indicating antagonism between canonical and non-canonical Wnt pathways in regeneration control. Moreover, Lrp6 co-receptor deletion enhances CM proliferation from neonatal to adult stages and post-MI regeneration through the ING5/P21 pathway. Exploiting this pathway, AAV9-delivered miRNAi targeting *Lrp6* improves cardiac repair (Wu et al. 2021). In supporting that inhibition of Wnt promotes adult heart regeneration, dampening myocardial Wnt signaling by the endocardial Notch signaling promotes CM formation and zebrafish heart regeneration (Zhao et al. 2019).

In contrast, some studies have reported that activation of Wnt signaling promotes β-catenin degradation. GSK3β is a core regulator of the canonical pathway and promotes β-catenin degradation through phosphorylation. Genetic ablation of *Gsk-3β,* which activates Wnt signaling prevents post-MI remodeling and enhances stress-induced CM proliferation in adult hearts (Fan et al. 2018). Pharmacological inhibition of GSK3β using CHIR99021 elevates EdU ^+^/Ki67 ^+^ CM populations and promotes human atrial myocyte proliferation (Wang et al. 2016). Combined GSK3β inhibition and reduced cell-cell contact enable a 100 - to 250-fold expansion of human iPSC-derived CMs (hiPSC-CMs) in vitro. This expansion is mediated by LEF/TCF activity and AKT phosphorylation, preserves cellular functionality, and enables scalable drug screening platforms (Buikema et al. 2020). Similarly, N-cadherin antibody treatment releases membrane-bound β-catenin, elevates cytoplasmic β-catenin levels, and activates Wnt signaling to enhance proliferation in mature CMs. Critically, this treatment promotes cardiac regeneration after ischemic injury in adult mice with N-cadherin overexpression (Fan et al. 2018; Tsai et al. 2025). The GSK3β inhibitor BIO exhibits analogous pro-proliferative effects in adult mammalian CMs, suggesting conserved mechanisms regulating stem cell self-renewal and differentiated CM proliferation (Tseng et al. 2006). Kruppel-like factor 1 (KLF1), a zinc finger transcription factor, epigenetically activates CM cell-cycle genes and Wnt/β-catenin signaling, maintaining proliferative capacity during regenerative phases but declining in adulthood (Hao et al. 2025; Yuce and Ozkan 2024). In addition, Wnt10b promotes coronary neovascularization via the NF-κB/VEGFR2 axis, accelerating capillary formation and reducing fibrosis in infarcted regions (Paik et al. 2015), suggesting effects of Wnt signaling promoting heart repair could be mediated by non-myocytes.

Some other signaling pathways or components might interact with Wnt signaling to regulate CM proliferation to improve cardiac regeneration. For example, Magadum et al. reported that induction of the pyruvate kinase M2 isoform (PKM2) upregulated *Ccnd1* and *c-Myc* by directly interacting with β-catenin, thereby promoting CM cell cycle re-entry to facilitate heart regeneration (Magadum et al. 2020); in contrast, Hauck et al*.* showed PKM2 knockout enhanced CM division through Akt-mediated β-catenin nuclear translocation (Hauck et al. 2021). The mechanism of PKM2 interaction with β-catenin is controversial and needs further exploration.

Although a general idea holds that inhibition of Wnt signaling is advantageous for heart repair and regeneration, a wealth of evidence on the dual regulatory roles of Wnt signaling in CM proliferation, heart repair, and regeneration makes it necessary to interpret the results very carefully and to consider the specific conditions,such as (1) developmental stage-specificity: canonical signaling drives embryonic CM precursor proliferation but is suppressed postnatally (Quaife-Ryan et al. 2020). (2) pathway antagonism: non-canonical pathways compensate via alternative mechanisms (e.g., JNK1/CREB1) (Liu et al. 2022; Peng et al. 2020). (3) multifactorial integration: metabolic shifts (PKM2), epigenetic regulation (KLF1), and structural dynamics (N-cadherin) collectively modulate regenerative outcomes (Hauck et al. 2021; Howson et al. 2017; Magadum et al. 2020; Tsai et al. 2025; Yuce and Ozkan 2024). (4) different injury manipulation and diverse regeneration stages. (5) Many signaling pathways (Notch, Hippo-YAP, TGF-β etc.) cross-talk with Wnt to influence the outcomes. (6) Wnt regulates non-myocytes (such as endothelial cells, macrophages) to modulate neovascularization and inflammation for heart regeneration.The role of the Wnt pathway in cardiac regeneration is remarkably complex, as it encompasses multiple homologs at various levels of the signaling cascade. Consequently, the pathway may yield distinct outcomes depending on the developmental stage. Furthermore, interventions targeting different components of the pathway or acting at distinct steps of Wnt signal transduction can exert bidirectional effects, either promoting or inhibiting CM proliferation. Therefore, Wnt signaling regulates CM proliferation and heart regeneration is complex and context-dependent, and requires further investigation.

### NRG1-ErbB signaling in cardiac development and regeneration

Neuregulin 1 (NRG1), a member of the epidermal growth factor (EGF) family, functions as a key regulator of cardiac development, structural integrity, and functional homeostasis in the adult heart by binding to ErbB tyrosine kinase receptors (ErbB1-4) (Wadugu and Kuhn 2012). Both NRG1 and its receptors—including ErbB2, ErbB3, and ErbB4—are essential for proper cardiac development. Mouse embryos deficient in either *Erbb2* or *Erbb4* receptors develop trabeculation defects and cardiac valve malformations. Furthermore, Gemberling et al. identified NRG1 as a potent CM mitogen, demonstrating that it can activate the cardiac regeneration process in zebrafish even in the absence of injury (Gemberling et al. 2015). In the heart, NRG1 initiates transmembrane signaling by binding to the ErbB4 receptor and forming a heterodimeric complex with ErbB2. This interaction triggers tyrosine phosphorylation within the intracellular kinase domain, recruiting downstream SH2 domain-containing signaling molecules such as those involved in the PI3K/AKT and MAPK/ERK pathways. These cascades subsequently modulate CM proliferation (Taniguchi et al. 2015), differentiation, and survival (Citri and Yarden 2006). Emerging evidence highlights the NRG-1/ErbB2/ErbB4 axis not only as essential for cardiac morphogenesis but also as a central regulatory network for post-injury regenerative repair (Bersell et al. 2009).

In adult zebrafish cardiac injury models, Nrg1 robustly activates CM mitosis via ErbB2 receptors (Gemberling et al. 2015). *Erbb2*, indispensable for embryonic myocardial proliferation, transiently reactivates in adult hearts to orchestrate phenotypic remodeling, downregulating mature CM markers (e.g., *Tnni3*, *Myh6*) while upregulating the expression of embryonic genes (e.g., *Nppa*, *Acta2*) . This transient expression, critical for initiating a regenerative program, is not sustained beyond the early postnatal period, thereby limiting the capacity for CM proliferation and regenerative repair in adulthood. This deficit can be rescued by administering recombinant NRG1 or a constitutively active ErbB2 (caErbB2), which promotes cell cycle re-entry and division of CMs in the injured zone, thereby facilitating repair (Bersell et al. 2009). Moreover, single-cell transcriptomics identified a glycolytic shift in proliferative border zone CMs, a metabolic reprogramming driven by the NRG1/ErbB2/HIF-1α axis, which was subsequently confirmed in the mammalian mouse heart (Honkoop et al. 2019).

In addition to its core mitogenic role, the NRG1-ErbB signaling axis engages in crosstalk with other pathways to exert multifaceted cardioprotective and regenerative effects. Beyond mitotic regulation, NRG1 counteracts H_2_O_2_-induced CM apoptosis by reducing reactive oxygen species (ROS) levels and alleviating endoplasmic reticulum stress through PI3K/AKT signaling, demonstrating dual cardioprotective effects (Xu et al. 2014). Exercise training enhances endogenous cardiac repair by upregulating *Nrg1* expression, which activates ErbB2/ErbB4 receptors and downstream PI3K/AKT signaling. This cascade promotes CM proliferation, survival, and neovascularization, thereby improving myocardial perfusion (Cai et al. 2016). Recombinant NRG1β attenuates cardiac fibrosis in remodeling and HF models, with normal human ventricular fibroblast lines exhibiting enhanced proliferation and viability upon NRG1β stimulation, suggesting direct antifibrotic and pro-regenerative actions (Kirabo et al. 2017). In summary, the NRG1-ErbB axis is a promising therapeutic target for cardiac regeneration. Its activation stimulates CM proliferation, fosters neovascularization, reduces fibrosis, and improves functional recovery, offering a multifaceted strategy against ischemic and degenerative cardiac pathologies.

### MAPK signaling in cardiac regeneration: dual roles and mechanistic insights

MAPKs, a family of serine-threonine protein kinases, regulate cardiovascular processes including cell proliferation, differentiation, stress responses, and inflammation through a three-tiered kinase cascade (MAPKKK-MAPKK-MAPK) activated by extracellular stimuli such as growth factors (e.g., FGF, PDGF) (Muslin 2008). Members of the MAPK family exhibit dynamic and often opposing roles in cardiac regeneration, balancing inhibitory and pro-regenerative functions.

#### Contrasting roles of p38 and ERK

P38 functions as a negative regulator of cardiac proliferation. Its active form suppresses mitosis-associated genes, impairing regeneration in zebrafish and mammalian CMs. Pharmacological inhibition or genetic knockdown of p38α improves ventricular function and reduces fibrosis (Jopling et al. 2012b). Additionally, p38 MAPK mediates CM apoptosis via the AMPK-p38-Bax cascade under ischemic conditions (Borutaite 2008). ERK acts as a central downstream effector, integrating upstream signals to drive CM proliferation. In adult zebrafish, localized H_2_O_2_ signaling generated after injury inactivates redox-sensitive Dusp6 phosphatases, relieving inhibition of the ERK1/2 pathway. Enhanced ERK1/2 phosphorylation synergizes with immune cell recruitment to promote regeneration (Han et al. 2014). Inhibition of *Dusp6* further amplifies cardiac repair by deregulating Ras/MAPK signaling, particularly ERK phosphorylation, even under restricted ErbB/PDGF receptor signaling (Missinato et al. 2018).

#### ERK as a multifunctional hub

ERK not only serves as the core of MAPK signaling but also amplifies ErbB2 activation through positive feedback. Transient overexpression of activated ErbB2 in CMs stimulates ERK signaling (Aharonov et al. 2020). In mice, ErbB2-mediated NRG1 signaling is essential for postnatal regeneration, as caErbB2 drives CM dedifferentiation and proliferation via ERK/AKT/GSK3β/β-catenin activation. Notably, caErbB2 induces pathological hypertrophy in uninjured hearts while promoting post-infarction repair (Bersell et al. 2009). Similarly, the caErbB2 mutant potently enhances proliferation in hiPSC-CMs and neonatal rat ventricular myocytes through ERK-dependent mechanisms, reversible by ERK inhibitors (Strash et al. 2021).

There are diverse ERK activation mechanisms for CM proliferation. For example, Agrin is a neonatal extracellular matrix glycoprotein that promotes CM dedifferentiation and proliferation in infarcted adult mice by binding α-Dystroglycan (DAG1) and activating ERK/YAP pathways (Bassat et al. 2017). In hiPSC-CMs, overexpression of N-cadherin enhances paracrine-mediated angiogenesis via ERK activation (Lou et al. 2020). Furthermore, Mitogen-Activated Protein Kinase Kinase (MEK) inhibitor AZD6244 treatment in a zebrafish ventricle resection model inhibits CM proliferation at injury sites, while pERK is induced in non-CMs during cardiac regeneration, and MEK1 dominant-negative mutants impair cardiac regeneration, highlighting ERK’s indirect role in endothelial cells in modulating the regenerative microenvironment (Liu and Zhong 2017). Synergistic suppression of MAPK and PI3K-AKT pathways accelerates structural and functional maturation of hiPSC-CMs, offering novel strategies for in vitro disease modeling (Garay et al. 2022).

The MAPK/ERK axis emerges as a pivotal regulator of cardiac regeneration, orchestrating CM reprogramming, neovascularization, and microenvironmental crosstalk. Its ability to integrate multidimensional signals positions ERK as a therapeutic linchpin for cardiac repair, offering novel avenues for regenerative interventions while deepening mechanistic understanding of heart regeneration.

### Notch signaling in cardiac development and regeneration

The Notch pathway, a highly conserved signaling system, regulates embryonic development, tissue regeneration, and mitochondrial metabolism through its core components: Notch receptors (Notch1-4), ligands (JAG1-2, DLL1-4), and downstream effectors (CSL (CBF1/Su (H)/Lag1) transcription factor complex) (Bray 2006). Signaling is initiated by ligand-receptor binding, triggering proteolytic cleavage of the NICD, which translocates to the nucleus to modulate target gene (such as *Hes/Hey*) expression (Bray 2006).

Notch plays an important role in cardiac regeneration. It was first confirmed in zebrafish, where adult zebrafish have a remarkable capacity for cardiac regeneration, in which the markedly increased expression of *notch1b* and *deltaC* is closely related (Raya et al. 2003). Using fluorescence reporter, fate mapping, and ventricle-specific ablation, it was found that ventricular injury activates Notch signaling in the atrial endocardium. Inhibition of Notch blocks atrial-to-ventricular transdifferentiation and cardiac regeneration (Zhang et al. 2013). In addition, there are multiple molecules in the zebrafish endocardium that promote CM proliferation, like serpine1 and Notch signaling (Münch et al. 2017). Following ventricular apex amputation in zebrafish, Notch receptor expression is specifically activated in the endocardium and epicardium (but not myocardium) to stimulate cardiac regeneration. However, excessive Notch signaling inhibits CM proliferation and impairs regeneration (Zhao et al. 2014). Beyond injury-induced Notch receptor upregulation in endocardium/epicardium, macrophages recruited to the epicardial-myocardial niche trigger epicardial dilatation and *vegfaa* upregulation, driving CM proliferation during zebrafish heart regeneration (Bruton et al. 2022). Concurrently, the Brg1-Kdm7aa-Notch axis in cardiac endothelial cells (including endocardium) promotes myocardial regeneration through enhanced H3K4me3 modification at Notch promoters (Xiao et al. 2023). Although acetylation stabilizes the NICD in neonatal rat CMs, amplifying its transcriptional activity to suppress apoptosis and sustain CM proliferation (Collesi et al. 2018), the pathway is unable to drive regeneration in the adult heart due to permanent epigenetic modifications to the promoter of the Notch response (Felician et al. 2014).

Similarly, notch signaling was activated after MI in rats and mice, and Notch signaling had a protective effect on the heart (Øie et al. 2010). Notch1 signaling was activated during myocardial ischemic preconditioning and ischemic postconditioning, and could enhance cell viability and inhibit apoptosis (Zhou et al. 2013). Notch1 signaling protects CMs from apoptosis by regulating Bcl-2/Bax expression and inhibiting caspase-9/−3 activation (Yu and Song 2014). Furthermore, Notch1 synergizes with the Keap1-NRF2 pathway to enhance neonatal CM activity, suppress apoptosis, reduce ROS formation, and boost antioxidant capacity (Zhou et al. 2020).

The Notch pathway integrates hemodynamic cues during cardiac regeneration. Following injury, hemodynamic changes induce endocardial *klf2a* expression via the mechanosensitive channel *trpv4*. Klf2a activates Notch signaling, which non-cell-autonomously initiates myocardial ErbB2 and BMP signaling to promote CM reprogramming and regeneration (Gálvez-Santisteban et al. 2019). Complementing this mechanotransduction pathway, primary cilia sense hemodynamic changes and induce *klf2a/klf2b* expression to activate endocardial Notch signaling. Both zebrafish *klf2* homologs are functionally equivalent in transducing mechanical cues and are essential for Notch activation and subsequent regeneration (Li et al. 2020a). A novel role for mechanical shear stress signaling in activating the Notch pathway and regulating cardiac regeneration is revealed.

Interestingly, some other regulators induced-heart regeneration could be mediated by Notch. For example, Exogenous high-mobility group box 1 protein upregulates cardiac regeneration genes and enhances c-kit + cardiac progenitor cell proliferation via Notch activation, and these regeneration effects could be abolished by the Notch inhibitor DAPT (Limana et al. 2013). It has been reported that Neurite outgrowth inhibitor-B (Nogo-B) promotes angiogenesis and improves cardiac repair after MI, whereas Notch1 deficiency abolishes these benefits (Gao et al. 2015; Zheng et al. 2022), suggesting that Notch1 mediates the effect of Nogo-B, and the Nogo-B -Notch1 axis could be a potential target for ischemic heart disease therapy. In transgenic mice overexpressing Jagged1 in myocytes, sustained cardiac precursor and myocyte proliferation after birth, while in adult hearts under pressure overload, Notch activation balances fibrotic remodeling and repair by suppressing CM hypertrophy, TGF-β/CTGF-mediated fibrosis, and myofibroblast proliferation while expanding *Sca-1*^*( +)*^*/Nkx2.5*^*( +)*^  precursors (a population of cardiac progenitor cells marked by stem cell antigen-1 and the cardiac transcription factor NK2 homeobox 5) (Nemir et al. 2014).

In addition, there are mechanistic synergistic and antagonistic effects of Notch signaling pathway by interactions with other signaling. For example, BMP (bone morphogenetic protein) interacts with Notch signaling pathway at multiple levels during development, tissue homeostasis and regeneration. BMP Crosstalk: Beyond activating Notch via hemodynamic sensing and coordinating BMP/ErbB2 signaling for CM reprogramming, BMP acts downstream of Notch during ventricular regeneration to differentially influence CM cell-cycle phases (Liang et al. 2015; Wang et al. 2021a). Wnt Antagonism: Regenerating zebrafish hearts show reduced expression of Wnt antagonists wif1 and notum1b. This downregulation enhances endocardial Notch signaling, which promotes CM proliferation by suppressing myocardial Wnt activity (Munch et al. 2017; Zhao et al. 2019). Akt Synergy: Notch1 enhances Akt activity, stabilizing mitochondrial membrane potential and reducing oxidative stress to mitigate ischemia–reperfusion injury (Gude et al. 2008).

While Notch critically regulates post-MI neovascularization and inflammation (e.g., Notch1 deficiency exacerbates infarction (Li et al. 2011)), its regenerative potential is constrained in adult mammals by epigenetic silencing. Overcoming this barrier, through reactivation of Notch signaling or reversal of terminal CM differentiation, remains a pivotal challenge for achieving functional cardiac regeneration.

As discussed above, while each key pathway (e.g., Hippo, Wnt, NRG1-ErbB, MAPK, and Notch) plays a critical role in cardiac regeneration, it is important to note that crosstalk among these pathways can be either synergistic or antagonistic. And each pathway and the crosstalk networks are dynamic during different stages of heart regeneration. However, more studies should be performed to reveal this complex crosstalk and application for efficient heart regeneration in the future.

### Crosstalk of multiple signaling pathways in cardiac regeneration

As the key effector and central signaling integrator of the Hippo pathway, YAP coordinates cellular processes by orchestrating crosstalk with multiple pathways. YAP engages in well-established crosstalk with Wnt signaling: it forms a transcriptional complex with β-catenin to enhance canonical Wnt activity, and it transcriptionally upregulates *Wls* to mediate non-canonical Wnt signaling that suppresses fibroblast activation. YAP also intersects with NRG1–ErbB4 signaling and activates insulin-like growth factor (IGF) signaling to promote cardiac growth. (Liu et al. 2021; Haskins et al. 2014; Xin et al. 2011)

Wnt signaling itself participates in dual modes of crosstalk. Its β-catenin-independent branch, mediated by WNT5A, coordinates with the cell division machinery to promote cytokinesis (Fumoto et al. 2012). Additionally, crosstalk between Notch and Wnt signaling has been revealed in zebrafish, where endocardial Notch activation following injury induces Wnt inhibitors, and Wnt inhibition can compensate for impaired Notch function in CM proliferation (Zhao et al. 2019).

Notch signaling engages in crosstalk with multiple pathways as well. Through Notch1/Hes1 signaling, it inhibits PTEN, thereby activating the PI3K/AKT pathway to support cell survival. In the developing heart, Notch activation upregulates *Efnb2*, which in turn promotes *Nrg1* expression—establishing crosstalk with the ErbB signaling pathway to drive trabecular cardiomyocyte differentiation.

The PI3K/AKT axis emerges as a central node where multiple signaling pathways converge. This axis receives inputs from Notch1/Hes1 (via PTEN regulation), VEGF/VEGFR2, NRG1/ErbB4, and TWEAK. Melatonin also interfaces with this network through Notch1/Hes1-dependent modulation.

Beyond these pairwise interactions, higher-order coordination is achieved through multi-target regulation. A cocktail of five small molecules (5SM), which consists of phenylephrine hydrochloride (α1-adrenergic receptor agonist), baricitinib (JAK1 inhibitor), harmine (DYRKs inhibitor), VO-Ohpic trihydrate (PTEN inhibitor), and AZD3965 (MCT1 inhibitor), simultaneously engages these distinct nodes to induce a metabolic shift toward glycolysis, promoting CM dedifferentiation and cell-cycle re-entry.

Furthermore, this metabolic reprogramming is intimately linked to cellular redox biology: reduced mitochondrial activity lowers ROS production, thereby alleviating ROS-induced DNA damage and cell-cycle arrest. Conversely, the post-infarct microenvironment is characterized by excessive ROS, which drives oxidative stress, promotes pathological remodeling, and suppresses CM proliferation. Given the extensive crosstalk among pathways such as PI3K/AKT, Wnt, and Hippo, single-target interventions may face limited efficacy or off-target toxicity. Multi-target pharmacological strategies, such as complex formulations or natural multi-active compounds, offer the potential to synergistically modulate the pro-regenerative microenvironment by simultaneously regulating oxidative stress, metabolic reprogramming, and cell-cycle machinery, providing a more integrated framework for cardiac regeneration. For instance, the combination of CHIR99021 and A-485 induces CM dedifferentiation into regenerative cells (Zhou et al. 2024), while dual inhibition of TLR4 signaling with cPIP2 and rosuvastatin synergistically improves post-MI cardiac function (Lee et al. 2025).

## Cellular microenvironment signaling regulates cardiac regeneration

**Fig. 2:**
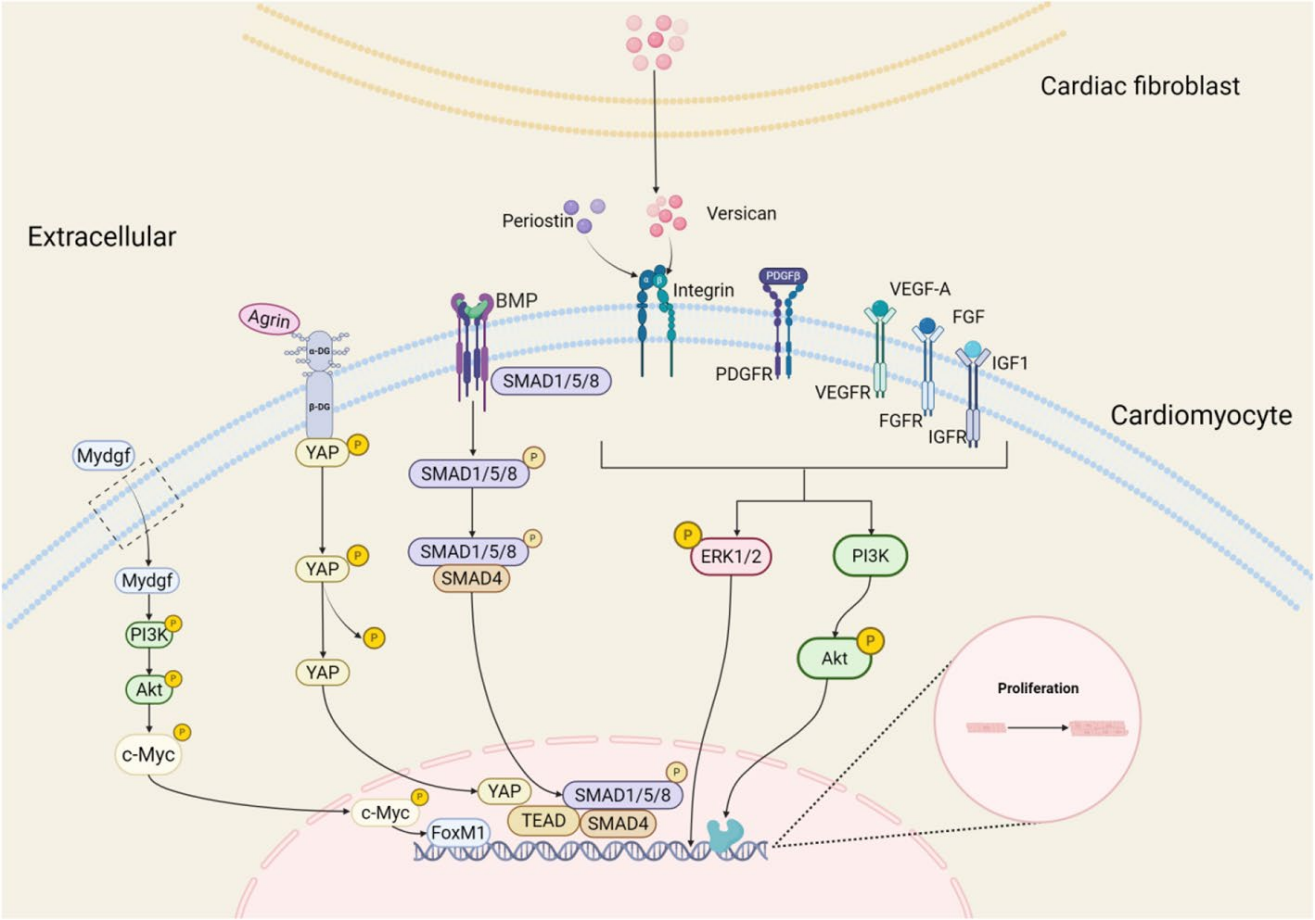
Mechanisms of signaling pathways associated with cellular microenvironmental regulation of cardiac regeneration. Created in https://BioRender.com

Except the key signaling pathways discussed above, recent progress has demonstrated that cellular microenvironment signaling, such as extracellular matrix (ECM) proteins and growth factors, is also involved in heart repair and regeneration (Fig. 2).

### Extracellular matrix

The ECM constitutes a sophisticated three-dimensional network within the extracellular space, primarily composed of fibronectin, glycosaminoglycans, proteoglycans, and mucoid substances. This dynamic architectural framework establishes a biochemically active microenvironment that critically regulates cellular processes, including proliferation, differentiation, migratory behavior, adhesion dynamics, and regeneration. (Wang et al. 2024b)

In the context of cardiac regeneration, the ECM not only provides mechanical support for the heart but also dynamically regulates molecular signaling for inflammatory responses, cell proliferation and migration, thereby driving tissue repair (Frangogiannis 2017). For example, TLR4/2 signaling activation stimulates matrix metalloproteinase (MMP) expression and proinflammatory factor release, thereby facilitating ECM degradation and subsequent reconstruction, whereas TLR deficiency inhibits fibrosis and improves cardiac function (Oyama et al. 2004). Functioning as the central mechanotransducer, YAP translates the mechanical softening – induced by exogenous fetal ECM and mechanical unloading and sensed via cytoskeletal remodeling – into the activation of CM cell-cycle activity and subsequent cardiac regeneration (Mercer et al. 2013; Wang et al. 2020a).

ECM-immune interactions further shape cardiac repair dynamics. Early post-injury, neutrophils and macrophages infiltrate the site, with inflammatory mediators inducing MMP production to degrade ECM components (Jugdutt 2003). Phagocytic clearance of cellular debris then triggers anti-inflammatory mediator release, transitioning the microenvironment toward repair via ECM deposition (Frangogiannis 2017). Fibroblasts contribute through ECM synthesis and myofibroblast differentiation, while regulatory T cells modulate remodeling by polarizing macrophages toward M2 phenotypes, balancing inflammation and enhancing reparative signaling (Zouggari et al. 2009). Zebrafish studies reveal that collagen reduction, combined with specific ECM protein surges during early regeneration, may recruit macrophages via growth factor release, establishing pro-proliferative signaling networks (de Preux et al. 2016; Han et al. 2015).

Emerging biomimetic strategies leverage ECM properties for therapeutic innovation. Hydrogels mimicking ECM mechanics offer dual functionality as mechanical supports and drug/cell delivery systems to modulate infarct microenvironments (He et al. 2023). Wang et al*.* demonstrated that neonatal mouse decellularized ECM reduces matrix stiffness to activate cytoskeletal polymerization and YAP signaling, thereby enhancing angiogenesis and attenuating ventricular remodeling (Wang et al. 2020a). Belviso et al. optimized a sodium dodecyl sulfate /Triton X-100 decellularization protocol for human left ventricular samples, producing bioactive 3D scaffolds (d-ECM) with preserved structural-protein composition after antimicrobial and UV sterilization (Belviso et al. 2022). Wang X et al*.* further engineered injectable cardiac-derived dECM particles that stabilize bioactive molecule release, improving cardiac function and mitigating ventricular remodeling in murine MI models (Wang et al. 2022a).

Combinatorial approaches integrating ECM scaffolds with cellular therapies show enhanced efficacy. Jiang et al*.* reported that porcine small intestinal submucosal ECM combined with human cardiovascular progenitor cells (hCVPCs) and CMs (hCMs) synergistically enhanced CM proliferation and graft survival in infarcted murine hearts. This effect was driven by optimized collagen III/I ratios and hCVPC-hCM paracrine interactions that upregulated repair-associated proteins (Jiang et al. 2023). Chen et al*.* demonstrated zebrafish ECM-mediated cardiac functional recovery in adult MI mice via ErbB2 pathway activation, inducing residual CM proliferation and cardiac precursor cell chemotaxis, with parallel mitogenic effects observed in human precursors in vitro (Chen et al. 2016). Additionally, Liu et al*.* developed cardiac fibroblast-derived ECM-modified silk fibroin scaffolds that promote brown adipose stem cell differentiation into CMs through β1-integrin-dependent TGF-β1 signaling (Liu et al. 2020). Recent research has found that The pathological degradation of cardioprotective heparan sulfate proteoglycan (HSPG) by LYZ2-overactivated lysosomal activity in endocardial cells exacerbates cardiac injury, whereas LYZ2 knockout rescues HSPG levels, thereby attenuating CM apoptosis and improving post-infarction repair (Fan et al. 2025).

Collectively, these advances highlight the therapeutic potential of multidimensional ECM modulation, integrating mechanical cues, signaling activation, and cellular crosstalk to reconstruct pro-regenerative microenvironments, thereby advancing novel strategies for cardiac repair. Moreover, some important components of the ECM proteins have recently been shown to regulate cardiac regeneration. The following will briefly introduce Agrin, Follistatin like protein 1 (FSTL1), Periostin and Versican in regulating cardiac repair.

#### Agrin-DGC

Agrin, a HSPG, constitutes a critical component of the ECM. The dystrophin glycoprotein complex (DGC) is a multimeric transmembrane assembly predominantly localized in CM membranes. Agrin binds to dystroglycan and dystrophin to stabilize the DGC, thereby disrupting the inhibitory function of DGC on CM proliferation and modulating downstream signaling pathways.

Under physiological conditions, DGC sustains CM terminal differentiation by sequestering the transcriptional co-activator YAP, thereby preventing its nuclear translocation and subsequent activation of pro-proliferative gene programs (Morikawa et al. 2017).

Notably, post-injury cardiac microenvironments exhibit a paradigm shift: endogenous upregulation or therapeutic administration of Agrin competitively displaces Yap from DGC complexes. This liberation facilitates Yap nuclear translocation, reinitiating cell cycle progression in mature CMs (Bassat et al. 2017). Murine model investigations corroborate this mechanism, revealing that Agrin administration not only induces CM dedifferentiation and proliferation but also enhances tissue remodeling through two synergistic pathways: 1) integrin-mediated cytoskeletal reorganization that restores electromechanical coupling, and 2) paracrine modulation that suppresses inflammatory responses and pathological fibrosis (Bassat et al. 2017; Bigotti et al. 2020). These multifaceted effects collectively promote functional myocardial regeneration in lesioned areas (Baehr et al. 2020).

Despite these promising therapeutic implications, critical knowledge gaps impede clinical translation. Current research priorities involve elucidating the structural dynamics of Agrin-DGC interactions through advanced techniques, such as single-cell transcriptomics and intravital imaging. Concurrently, developing ECM-targeted delivery systems remains imperative to optimize the therapeutic window between regenerative activation and fibrotic suppression (Bigotti et al. 2020).

#### FSTL1

FSTL1, an ECM protein secreted by cardiac fibroblasts, plays a key role in cardiac injury repair. Its expression is acutely induced after MI. FSTL1 maintains cardiac structural integrity and prevents rupture by promoting fibroblast activation, proliferation, and ECM synthesis (Maruyama et al. 2016). Studies have shown that the low-glycosylated form of FSTL1 (e.g., synthesized by bacteria or modified by mutations at the N180Q site (Magadum et al. 2018)) is able to specifically stimulate CM proliferation after hypoxia, whereas mammalian sources of glycosylated FSTL1 have restricted activity and their post-translational modifications are important in regulating their function. In terms of specific mechanisms, FSTL1 exerts cardioprotective effects. It reverses the abnormal metabolic phenotype of reduced fatty acid oxidation and enhanced glycolysis in HF by activating AMPK to regulate energy metabolism (Ogura et al. 2012). It is important to note that epicardial-derived FSTL1 is critical for cardiac regeneration, and endogenous myocardial FSTL1 is ineffective at promoting repair, whereas exogenous epicardial FSTL1 administration significantly enhances CM proliferation and improves survival (Wei et al. 2015). Its expression is regulated by AKT protein kinase and reduces CM death in ischemic injury through the autocrine/paracrine pathway (Oshima et al. 2008). In addition, local delivery of FSTL1 by bioengineered patches significantly reduced scar formation, improved ventricular remodeling, and increased ejection fraction in an animal model of MI, while promoting neovascularization and CM regeneration (Altekoester and Harvey 2015).

#### Periostin

Periostin, encoded by the *POSTN* gene, functions as a multifunctional matricellular protein to reactivate cell cycle progression in terminally differentiated CMs through integrin receptor-mediated activation of the PI3K/AKT signaling axis (Kuhn et al. 2007). In acute MI models, periostin expression was significantly upregulated and coordinated structural preservation via fibroblast recruitment and collagen matrix reorganization, with genetic ablation exacerbating myocardial compliance abnormalities and ventricular rupture risk. Conversely, therapeutic overexpression demonstrates cardioprotective efficacy by enhancing tensile strength and mitigating rupture incidence (Oka et al. 2007). The fibrogenic activity of periostin is mechanistically linked to Scleraxis, a transcription factor that directly binds the *Postn* promoter to drive collagen biosynthesis. Genetic disruption of Scleraxis markedly attenuates pathological collagen deposition and maladaptive remodeling (Nagalingam et al. 2022). Post-MI, periostin plays a critical role in enhancing the healing process of the heart by promoting fibroblast recruitment and collagen fiber formation. Deficiency of periostin leads to impaired cardiac healing, as evidenced by reduced myocardial stiffness and increased risk of cardiac rupture, and its gene transfer effectively rescues these defects (Shimazaki et al. 2008).

Notably, periostin’s therapeutic profile demonstrates context-dependent variability. Oka et al*.* (2007) observed intact cardiac repair capacity in *Postn* knockout mice post-injury, implying microenvironmental compensation through redundant ECM signaling pathways. This paradoxical phenomenon may also stem from experimental model differences (e.g., type of injury, species specificity, or timing of intervention), such that Periostin regulates cardiac development by driving Epithelial-Mesenchymal Transition in the embryo, whereas in the adult heart it responds predominantly to acute injury signaling, and its function may be partially compensated by other ECM components (Conway and Molkentin 2008).

#### Versican

Versican (Vcan) is a cardiac fibroblast-derived ECM component. It consists mainly of polysaccharides and proteins with complex structures and multiple biological functions, and is a member of the hyalectan family of large chondroitin sulfate proteoglycans. Barallobre-Barreiro et al*.* found that in patients with HF, the accumulation of Versican and its specific cleavage product, Versikine, was associated with cardiac function impairment (Barallobre-Barreiro et al. 2021). Feng et al*.* showed that intramyocardial administration of Vcan has therapeutic potential to improve cardiac function in adult mice with MI. And Vcan had a proliferative effect on hiPSC-derived CMs. Mechanistically, Vcan activated integrin β1 and its downstream signaling, including ERK1/2 and AKT, thereby promoting CM proliferation and cardiac repair (Feng et al. 2024).

### Growth factor

Except ECM, the alterations of growth factors derived from different cells in the local environment also contribute to cardiac repair and regeneration. These growth factors include platelet-derived growth factor (PDGF), fibroblast growth factor (FGF), Myeloid-derived growth factor (Mydgf), Vascular endothelial growth factor (VEGF), IGF and so on (Table 1).

**Table 1:** Relevant growth factors regulating CM proliferation

Growth Factors	Regulatory mechanism	Reference
PDGF	Regulates angiogenesis via PI3K/AKT pathway	Lien et al. 2006
FGF	FGF1 enhances CM survival by activating the FGFR/ERK signaling pathway; FGF10, on the other hand, inhibited fibrosis and promoted CM renewal through the FGFR2b/PHLDA1/AKT axis	Zhou et al. 2023
MYDGF	Targeting the c-Myc/FoxM 1 pathway to stimulate CM regeneration	Wang et al. 2020b
VEGF	VEGFR1/VEGFR2-mediated regulation of CM morphogenesis and contractility	Braile et al. 2020
IGF	The IGF signaling pathway promotes CM proliferation and regulates embryonic heart development by activating key molecules such as YAP and β-catenin	Yu et al. 2013
HGF	HGF in combination with IGF-1 enhances angiogenesis by promoting CM survival and regeneration	Wang et al. 2014
NGF	CM proliferation and reduction of apoptosis	Lam et al. 2012
BMP7	Activation of CM proliferation through BMPR1A/ACVR1 and ACVR2A/BMPR2 receptors and their downstream SMAD5, ERK and AKT signaling pathways	Bongiovanni et al. 2024
Follistatin	Activation of the Hippo/YAP signaling pathway promotes endogenous CM proliferation	Wei et al. 2025
Angiopoietin-1	Enhancement of pERK1/2 signaling and inhibition of Bax protein	Tao et al. 2021

#### PDGF

The PDGF family demonstrates isoform-specific regulatory duality in cardiac regeneration, mediated through differential receptor binding and downstream signaling cascades. Experimental evidence identifies Pdgf-B as a critical mitogen in zebrafish cardiac repair, enhancing post-injury CM proliferation via Pdgfrβ-dependent DNA synthesis, with PDGFR pathway inhibition markedly impairing regenerative capacity (Lien et al. 2006). Parallel investigations reveal PDGF-C and PDGF-D isoforms modulate ECM homeostasis through coordinated PDGFRα/β activation, balancing synthetic and catabolic processes during tissue remodeling (Lee and Li 2018). This regenerative potential, however, coexists with inherent fibrotic risks. Murine models illustrate divergent pathological outcomes: PDGF-A overexpression induces pathological cardiac mesenchymal activation through PDGFRα, driving maladaptive ECM accumulation and fatal HF, whereas PDGF-B overexpression elicits localized fibrosis accompanied by compensatory hypertrophy (Gallini et al. 2016). Such paradoxical effects highlight the signaling precision required for therapeutic targeting. Mechanistic studies further demonstrate PDGFRα-mediated PI3K signaling maintains fibroblast homeostasis, with conditional deletion of PDGFRα in cardiac fibroblasts resulting in fibroblast depletion and disruption of the basement membrane and microvasculature (Ivey et al. 2019). The dual regulatory capacity of PDGF signaling—facilitating protective repair while potentiating fibrotic injury—necessitates nuanced therapeutic strategies in myocardial ischemia–reperfusion injury. Current challenges involve optimizing isoform-specific modulation to harness the regenerative potential while circumventing profibrotic cascades.

#### FGF

The FGF family exerts coordinated therapeutic effects on cardiac repair through multifaceted mechanisms involving CM proliferation, antiapoptotic signaling, and antifibrotic regulation. FGF1 enhances CM survival via FGFR-dependent ERK pathway activation, and preclinical studies have shown that intramyocardial injection of FGF1-loaded PLGA and PEG-PLGA microparticles similarly improves post-MI ejection fraction, angiogenesis, and arteriogenesis in rodent models, with PEG coating conferring no additional benefit over PLGA MPs alone (Pascual-Gil et al. 2017). In contrast, FGF10 attenuates myocardial injury through the FGFR2b/PHLDA1/AKT axis while concurrently suppressing fibrotic remodeling and stimulating CM renewal (Zhou et al. 2023). Functional divergence among FGF isoforms is further dictated by spatiotemporal expression patterns: embryonic FGF16, transcriptionally regulated by GATA4, drives CM proliferation during cardiac development and injury repair (Yu et al. 2016), whereas postnatally abundant FGF20 counteracts oxidative stress and pathological hypertrophy via ErbB2 receptor activation (Chen et al. 2024b).

Beyond isoform-specific functions, advances in biomaterial engineering address inherent pharmacokinetic challenges of FGF therapy. A notable innovation includes K2 (a peptide Lys-Lys-Pro-Leu-Gly-Leu-Ala-Gly-Phe-Phe) micelle-encapsulated bFGF developed by Wang et al*.*, which achieves ischemia-specific release and prolonged myocardial retention through an assembly-induced retention mechanism. This strategy significantly enhances contractile function while reducing fibrotic burden in preclinical evaluations (Wang et al. 2022b). These findings not only delineate the therapeutic potential of individual FGF isoforms but also underscore the pivotal role of engineered delivery platforms in overcoming limitations such as rapid clearance and off-target effects inherent to growth factor therapies.

#### MYDGF

Myeloid-derived growth factor (Mydgf) emerges as a pivotal regulator of post-MI repair, offering therapeutic potential through its multimodal actions on CM preservation and vascular remodeling. Mechanistically, Mydgf—primarily secreted by endothelial cells in ischemic myocardium—activates the c-Myc/FoxM1 axis to directly stimulate CM proliferation while concurrently promoting neovascularization, thereby synergistically attenuating infarct expansion and adverse cardiac remodeling (Wang et al. 2020b). Further investigations reveal its cardioprotective role in calcium homeostasis: Mydgf potentiates sarcoplasmic/endoplasmic reticulum calcium ATPase expression via PIM1 proto-oncogene upregulation, mitigating pressure overload-induced HF (Korf-Klingebiel et al. 2015). Genetic ablation studies corroborate these findings, demonstrating exacerbated fibrosis and functional decline in Mydgf-deficient mice post-MI. Conversely, exogenous recombinant Mydgf administration significantly reduces scar burden and restores contractile performance, underscoring its translational relevance (Korf-Klingebiel et al. 2015). These dual mechanisms—enhancing CM survival while orchestrating microvascular regeneration—position Mydgf as a promising candidate for ischemic heart disease therapeutics.

#### VEGF

VEGF demonstrates multifaceted regulatory capacities in cardiac regeneration, positioning it as a pivotal therapeutic target in cardiovascular medicine. Preclinical studies utilizing immunoliposome-encapsulated VEGF for MI targeting reveal enhanced neovascularization within ischemic regions, accompanied by improved contractile performance and ventricular fractional shortening (Formiga et al. 2010). Mechanistically, VEGF-A binds VEGFR1 and VEGFR2 to orchestrate CM morphogenesis, contractile optimization, and tissue repair. Notably, CM-derived VEGF-A is dynamically upregulated in response to pathological stimuli (e.g., inflammatory factors and mechanical stress), and elevated levels of its expression correlate with adverse clinical outcomes in cardiovascular diseases (Braile et al. 2020). Conversely, VEGF-A modRNA mediates transient, non-integrating VEGF-A expression in the myocardium (Carlsson et al. 2018). In murine and porcine MI models, a single administration of VEGF-A mRNA improves systolic function, enhances angiogenesis, and fibrosis, thereby prolonging survival (Zangi et al. 2013). These benefits derive in part from the expansion and cardiovascular specification of endogenous epicardial progenitors, as well as from VEGF-A delivery, which supports sustained cardiac repair with no immunogenicity or genomic integration.

Intriguingly, comparative analyses in zebrafish models unveil context-dependent duality: while endogenous *vegfaa* drives CM proliferation and angiogenesis during cardiac regeneration, its supraphysiological overexpression paradoxically impairs reparative processes at injury sites (Karra et al. 2018). These findings underscore the necessity of precise VEGF modulation, balancing pro-regenerative angiogenesis against potential maladaptive signaling cascades.

#### IGF

The IGF family exhibits multifaceted regulatory functions in cardiac regeneration, orchestrating cellular proliferation, developmental programming, and stress adaptation. Central to embryonic cardiogenesis, the IGF signaling axis orchestrates YAP and β-catenin activity to drive CM progenitor expansion. Complementarily, zebrafish models reveal that injury-induced *igf2b* upregulation serves as a critical regulator of CM division during cardiac repair (Huang et al. 2013). Intriguingly, Xin et al*.* (2011) identified YAP as a nodal regulator of IGF-mediated control over CM proliferation and embryonic heart size. Functional diversity emerges across IGF isoforms: IGF-1 counteracts hyperglycemia-induced cell-cycle arrest via β-catenin activation, enhancing CM survival under metabolic stress (Yu et al. 2013), while IGF2 operates as a paracrine effector in neonatal mice, facilitating regeneration through diploid mononuclear CM activation (Shen et al. 2020). However, this signaling cascade is tightly modulated by contextual factors. Wang et al*.* (2019b) revealed that embryonic programs (e.g., *IGF2BP3*) collaborate with immune mediators to promote proliferative responses in regenerating hearts, whereas acute stress suppresses IGF signaling and impairs reparative capacity. Although IGF-1/HGF co-administration improves post-infarction angiogenesis and cytoprotection, its efficacy in functional myocardial restoration remains constrained (Wang et al. 2014).

These findings underscore the therapeutic duality of IGF modulation—while its targeted activation synergizes with cellular therapies to optimize cardiac repair, precise spatiotemporal control is imperative to balance regenerative potential against pathway oversaturation.

#### Other growth factors

There are many other growth factors that play a role in cardiac regeneration. Hepatocyte growth factor (HGF) demonstrates cardioprotective efficacy in ischemia–reperfusion injury, where endogenous neutralization exacerbates CM apoptosis and infarct expansion. Conversely, exogenous HGF administration attenuates myocardial damage through dual mechanisms: 1) c-Met receptor-mediated suppression of apoptotic cascades, and 2) synergistic activation of IGF-1 signaling to enhance angiogenesis and CM survival (Nakamura et al. 2000; Wang et al. 2014). Lam et al*.* showed that Nerve growth factor (NGF) induces regenerative responses in zebrafish HF models by directly stimulating CM proliferation (Lam et al. 2012). Additionally, bone morphogenetic protein 7 (BMP7) coordinates CM renewal via multireceptor complexes (BMPR1A/ACVR1 and ACVR2A/BMPR2), activating SMAD5-ERK-AKT signaling cascades. Genetic inhibition of *BMP7* ablates regenerative capacity in zebrafish and neonatal mice, while its therapeutic supplementation enhances post-infarction repair across species (Bongiovanni et al. 2024). And hiPSC-CM spheroids (KO/OE hiPSC-CM) expressing HLA class I/II by knockdown and overexpressing *CCND2* secrete Follistatin, which activates the Hippo/YAP signaling pathway, significantly promotes the proliferation of endogenous CMs, and treats porcine myocardial ischemia/reperfusion injury (Wei et al. 2025). Moreover, enhancement of pERK1/2 signaling and inhibition of Bax protein by transient overexpression of Angiopoietin-1 (Ang-1) in atrial hiPSC-CMs resulted in survival and sustained secretion of Ang-1 in hypoxic environments and significantly ameliorated ventricular dilatation, promotion of proliferation of both host and donor CMs, and small arteriogenesis in a rat MI model after transplantation (Tao et al. 2021).

These findings underscore the therapeutic potential of engineered growth factor delivery systems. Spatiotemporal control over combinatorial factor release—particularly through biomaterial-mediated targeting—synergistically enhances endogenous repair mechanisms while minimizing off-target effects. Such precision approaches may overcome current limitations in achieving functional myocardial restoration, positioning growth factor cocktails as pivotal components of next-generation regenerative strategies.

## Cellular metabolic signaling regulates cardiac regeneration

### Cellular metabolism and hypoxia

Cellular metabolism is intimately involved in cardiac regeneration. After birth, the energy metabolism of CMs shifts from glycolysis to fatty acid oxidation, a change that is accompanied by an increase in mitochondrial ROS, which leads to DNA damage and promotes cell cycle arrest in CMs, resulting in the loss of regenerative capacity (Cardoso et al. 2020b). Recent data show that altering the metabolic enzymes PKM2, LDHA (lactate dehydrogenase A), PDK4 (pyruvate dehydrogenase kinase 4), SDH (succinate dehydrogenase), CPT1b (carnitine palmitoyl transferase 1b), or HMGCS2 (3-hydroxy-3-methylglutaryl-CoA synthase 2) is sufficient to partially reverse metabolic reprogramming (phenotypic alteration of glucose, fatty acid, and amino acid metabolism) and promotes adult CM proliferation (Chen et al. 2024a). Moreover, the hypoxic environment inhibits aerobic respiration and reduces ROS production and oxidative DNA damage, thereby reactivating the ability of CMs to divide and regenerate (Nakada et al. 2017). In addition, HIF-1α was able to maintain the regenerative capacity of CMs under hypoxic conditions (Kimura et al. 2015).

### Glucose metabolism

**Fig. 3:**
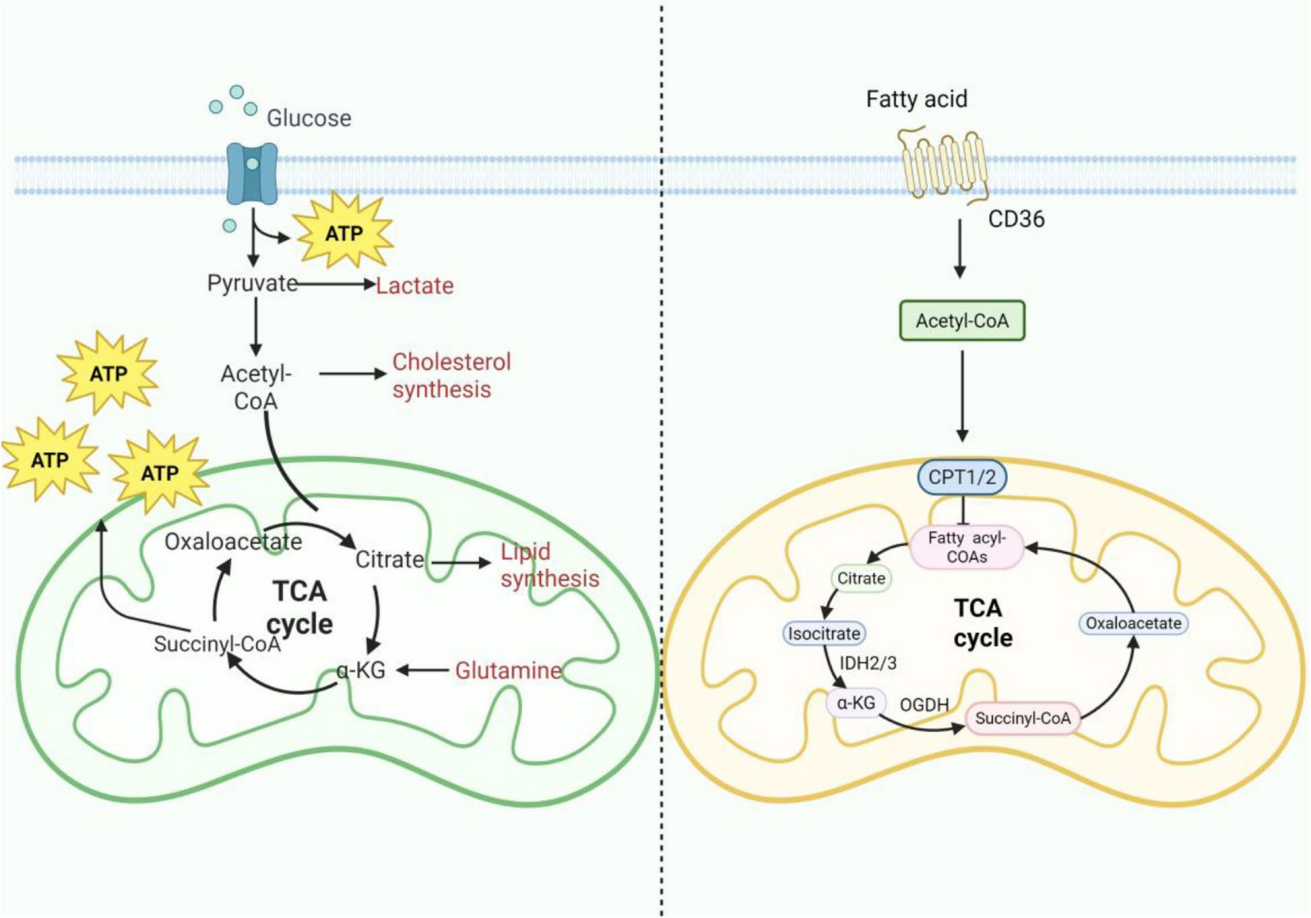
Metabolic shifts in cardiac regeneration. During the first week of life, the primary source of energy metabolism shifts from glycolysis to fatty acid beta-oxidation (FAO). Created in https://BioRender.com

Glycolytic metabolism emerges as a pivotal regulator of cardiac regeneration, orchestrating both bioenergetic and biosynthetic demands in proliferating CMs (Fig. 3). Fukuda et al*.* demonstrated that zebrafish myocardial repair involves injury-zone metabolic reprogramming via upregulation of *pkm/pdks*, promoting CM dedifferentiation through pyruvate dehydrogenase kinase (PDK)-mediated suppression of mitochondrial oxidation. Pharmacological inhibition of glycolysis or PDK activity significantly attenuates CM proliferative capacity, confirming the pathway’s necessity. Pharmacological inhibition of glycolysis or PDK3 significantly attenuates proliferative capacity, underscoring the pathway’s necessity (Fukuda et al. 2020). Complementary studies reveal that proliferating CMs in regenerating zebrafish hearts exhibit embryonic-like transcriptional profiles characterized by mitochondrial quiescence and glycolytic activation, a process governed by NRG1/ErbB2 signaling. Notably, ErbB2 activation induces analogous metabolic switching in murine CMs, enabling proliferation under stress conditions (Honkoop et al. 2019).

Mechanistic insights further identify glucose transport as a critical regulatory node. Cardiac-specific Glucose Transporter 1 (GLUT1) overexpression enhances glycolytic flux in neonatal mice, facilitating nucleotide biosynthesis and cryoinjury repair while suppressing fibrosis. This regenerative advantage is mediated by *Tnnt2*-low CM populations activated under hyperglycemic conditions (Fajardo et al. 2021). Parallel investigations demonstrate that moderate heart rate reduction enhances ATP biosynthesis efficiency and G1/S phase transition through glycolytic activation, improving regenerative outcomes in both physiological and post-injury states (Tan et al. 2022).

Key glycolytic enzymes exhibit dual regulatory roles:

(1) Checkpoint kinase 1 (CHK1) phosphorylates mTORC1/p70S6K to promote CM proliferation, with adenoviral overexpression restoring regenerative potential in adult infarcted hearts (Wei et al. 2024b). Hydrogel-delivered recombinant CHK1 activates PKM2 via Ser37/Tyr105 phosphorylation, driving glycolysis-mTOR crosstalk to enhance proliferation and mitigate inflammation (Cardoso et al. 2020a).

(2) PKM2 isoform dynamics govern metabolic fate: dimeric PKM2 accumulates glycolytic intermediates to modulate ATP-sensitive potassium channels and pentose phosphate pathway activation, supporting NADPH synthesis and stress adaptation (Jovanovic et al. 2005; Jovanovic and Jovanovic 2005; Magadum et al. 2020). Conversely, tetramer-stabilizing agents like TEPP-46 enhance oxidative metabolism and α-ketoglutarate production, partially reversing adult CM cell-cycle quiescence (Chen et al. 2024a; Zeng et al. 2024).

Recent advances by Nie et al. identify FOXK1/FOXK2 as transcriptional coordinators of metabolic-proliferative coupling (Cai et al. 2025). These factors activate CCNB1/CDK1 to drive G2/M transition while upregulating *Hif1α*-mediated glycolytic and pentose phosphate pathway reprogramming. Developmental decline in *Foxk1/Foxk2* expression correlates with regenerative capacity loss, whereas AAV9-mediated overexpression extends the proliferative window in adult infarcted hearts. This transcriptional-metabolic synergy opens new avenues for targeted regenerative therapies.

### Fatty acid metabolism

Fatty acid metabolism plays a paradoxical role in cardiac regeneration, balancing developmental maturation with retained proliferative capacity (Fig. 3). Unlike glycolytic-dependent neonatal CMs, adult mammalian hearts predominantly utilize fatty acid β-oxidation (Park et al. 2019), a metabolic shift coinciding with regenerative potential loss. Experimental disruption of FAO via CM-specific *Cpt1b* inactivation induces metabolic reprogramming, enhancing hypoxia tolerance and proliferation by overcoming maturation barriers (Li et al. 2023). Mechanistically, CPT1 inhibition downregulates *Parp1* expression, attenuating DUSP1 ADP-ribosylation to reduce p38 MAPK phosphorylation—thereby activating pro-proliferative signaling and improving post-infarction outcomes (Tang et al. 2025).

Developmental studies reveal FAO’s dual regulatory effects: PPARα-mediated β-oxidation transiently drives postnatal CM proliferation and hypertrophic maturation, with pathway inhibition delaying cell cycle exit without altering total CM numbers (Cao et al. 2019). Pharmacological modulation further demonstrates metabolic plasticity: carbacyclin activates PPARδ/PDK1/p308AKT/GSK3β/β-catenin to induce neonatal and adult CM proliferation, while *PPARδ* ablation impedes zebrafish heart repair (Magadum et al. 2017). Sphingolipid metabolism adds another layer of regulation—nuclear SphK2-generated S1P promotes CM proliferation via histone acetylation, whereas SphK1-derived S1P drives fibroblast fibrosis. Isoform-specific SphK modulation achieves synergistic repair by balancing regenerative and antifibrotic effects (Ji et al. 2024).

Metabolic-transcriptional crosstalk further orchestrates regeneration. *Foxp1* reprograms fatty acid-to-glycolytic metabolism through USP20/HIF1α/Hand1, with AAV9-mediated heart and neural crest derivatives expressed 1(*Hand1*) overexpression enhancing metabolic switching and functional recovery (Wang et al. 2025). CTBP2 senses NADH/NAD + and fatty acyl-CoA to regulate FOXO1-p21/p27, unlocking cell-cycle progression post-injury while maintaining quiescence under homeostasis (Meng et al. 2025). Glucocorticoid receptor (GR) suppresses glucose catabolism to enforce FAO-driven maturation; its inhibition reactivates glycolytic metabolism, promoting CM repopulation (Pianca et al. 2022).

Multi-omics analyses delineate dynamic postnatal metabolic remodeling: transient activation of branched-chain amino acid degradation and mevalonate/ketogenesis pathways synergizes with oscillating FAO activity to regulate regenerative decline. Key nodes include: mTORC1, enforcing proteomic shifts from glycolysis to FAO, with acute inhibition impairing proliferation despite unchanged protein synthesis (Paltzer et al. 2024). SCD1 suppresses induced CM reprogramming via PGC1α/PPARβ-mediated FAO inhibition; its knockdown enhances mitochondrial biogenesis (Jia et al. 2025). The sarcomere-metabolism axis functions through developmental isoform switching of troponin I — from skeletal troponin I (ssTnI) to cardiac troponin I — wherein sustained ssTnI expression promotes glycolysis and regeneration by rewiring metabolic-structural coupling (Aballo et al. 2023).

Collectively, these findings position FAO as both a developmental gatekeeper and therapeutic target. Strategic modulation of GR, mTORC1, SCD1-PGC1α/PPARβ, and mevalonate pathways may reverse metabolic maturation, offering novel avenues for regenerative intervention.

### Hypoxia

Hypoxia serves as a pivotal regulator of cardiac regeneration, with hypoxia-inducible factors (HIFs) acting as central mediators of oxygen-sensitive repair mechanisms. Composed of α/β heterodimers, HIF transcription factors dynamically orchestrate metabolic adaptation, inflammatory modulation, and tissue remodeling post-MI. Under hypoxic conditions, HIF-1α stabilization activates transcriptional programs governing glycolysis, angiogenesis, and cell cycle progression. Specifically, HIF-1α enhances glycolytic efficiency in CMs while synergizing with YAP/TAZ to promote neovascularization, thereby establishing metabolic and structural foundations for repair (Yuan and Braun 2017; Zhang et al. 2022).

Mechanistic studies reveal hypoxia’s pleiotropic effects across species: Neonatal rat models demonstrate HIF-1α/ERK-mediated upregulation of Jagged1 in CMs, driving cardiac stem cell differentiation via Notch1 signaling—a process abrogated by HIF or Notch inhibitors (Wang et al. 2018a). Zebrafish ventricular resection identifies hypoxia-induced dedifferentiation of mature CMs as a regenerative driver, with transcriptomic screens uncovering key regulatory clusters (Jopling et al. 2012a). Mammalian postnatal hearts exhibit oxygen-dependent cell cycle arrest, in which hyperoxia-induced ROS/DNA damage response (DDR) terminates proliferation. Hypoxic preconditioning, ROS scavenging, or DDR inhibition extends the regenerative window (Puente et al. 2014).

Systemic hypoxia interventions demonstrate translational potential: Sustained hypoxia (7% O₂) attenuates mitochondrial oxidative metabolism, reducing ROS-mediated DNA damage to reactivate adult murine CM proliferation and improve post-infarction contractility (Nakada et al. 2017; Nakada and Sadek 2021). CM-specific *Hif1α* knockout models reveal embryonic metabolic plasticity, compensating glycolytic deficiency through mitochondrial biogenesis, amino acid catabolism, and HIF2α/ATF4 signaling—confirming developmental independence from canonical *Hif1* pathways (Menendez-Montes et al. 2021).

Notably, HIF activation exhibits context-dependent duality. Chronic HIF-1α/HIF-2α stabilization precipitates pathological remodeling, including lipidosis, fibrosis, and metastatic cardiac tumors (Lei et al. 2008). These findings underscore the necessity for precise HIF modulation, balancing acute pro-regenerative signaling against chronic maladaptation. Therapeutic strategies targeting isoform-specific HIF dynamics or transient hypoxia mimicry may optimize cardiac repair while mitigating adverse outcomes.

## Transcription factors

Transcription factors (TFs) represent a class of coregulatory proteins that govern transcriptional programs by modulating chromatin accessibility, recruiting RNA polymerase, and binding specifically to *cis*-acting DNA elements within promoter or enhancer regions of target genes. Their functional networks establish the molecular framework for the spatiotemporal regulation of gene expression (Lambert et al. 2018). Functioning as "molecular switches" in cell fate determination and phenotypic plasticity, TFs not only integrate signaling inputs from pathways such as Wnt and Hippo-YAP to dynamically coordinate developmental and regenerative processes but also exhibit therapeutic potential for cardiac tissue repair. For instance, key regulators including GATA4, Tbx20, and Meis1 drive CM proliferation by synergistically activating cell cycle-associated genes while suppressing terminal differentiation signals. This dual regulatory capacity—balancing proliferative activation with differentiation arrest—positions transcriptional reprogramming as a pivotal strategy in cardiac regenerative medicine, offering novel avenues for post-injury myocardial repair (Table 2).

**Table 2:** Relevant transcription factors regulating CM proliferation

Transcription Factors	Experiments Models	Regulation mechanism in CM proliferation	Reference
Meis	CKO in mouse CM	Knockout Meis extends the proliferation window of CM and re-enters the cell cycle for mitosis	Mahmoud et al. 2013
Hoxb13	KO in mouse CM	Promoting CM mitosis after double knockout with the Meis gene	Nguyen et al. 2020
GATA4	KO in zebrafish CM	Regulation of paracrine factor FGF16 maintains cardiac repair capacity	Kikuchi et al. 2010
TBX20	KO in zebrafish CM	Induction of CM dedifferentiation and activation of BMP signaling pathway in endocardial cells promote CM proliferation	Fang et al. 2020
E2F		Regulation of cell cycle-related genes to influence CM proliferation	Wei et al. 2024a
Pitx2	CKO in mouse CM	Maintaining proper cardiac cell composition by maintaining proper mitochondrial structure and function	Tao et al. 2016
FoxM1	KO in zebrafish CM	Promoting CM regeneration through transcriptional regulation of cell cycle genes	Zuppo et al. 2023
KLF1	KO in zebrafish CM	Promoting CM proliferation and cardiac regeneration by regulating the Wnt/β-catenin signaling pathway	Ogawa et al. 2021
TP53	KO in zebrafish CM	Decreased TP53 levels promote CM proliferation	Shoffner et al. 2020
NRF1	KO in mouse CM	Nrf1 maintains cardiac homeostasis and regeneration by regulating the proteasome and antioxidant response pathways	Cui et al. 2021
NFYα	OE in mouse CM	Overexpression of NFYα significantly enhances CM division and promotes CM proliferation after cardiac injury	Cui et al. 2020
FoxO3	KO in neonatal mouse CM	FoxO3 regulates sFRP2 gene expression thereby inhibiting Wnt/β-catenin signaling activity thereby promoting CM proliferation	Xia et al. 2025
Foxp1	KO in mouse CM	Foxp1 deletion promotes CM proliferation by regulating the USP20-HIF1ɑ-Hand1 signaling pathway, and overexpression inhibits its proliferation	Wang et al. 2025
FoxK1/2	OE, CKO in mouse CM	Foxk1/2 promote CM proliferation through direct activation of cell cycle regulatory genes and indirect regulation of cell metabolism	Cai et al. 2025

### Meis and Hoxb13

Meis transcription factors, members of the Three-Amino-Acid-Loop-Extension homology domain family, serve as pivotal regulators of embryonic cardiac differentiation and orchestrate the CM cell cycle (Paige et al. 2012; Wamstad et al. 2012). Among these, Meis homeobox 1 (Meis1) activates target genes through cooperative binding with Hox transcription factors (Cesselli et al. 2001), while Meis2 and Meis3, sharing high structural homology with Meis1, exhibit distinct yet overlapping functional roles (von Burstin et al. 2010). Mechanistically, Mahmoud et al*.* (2013) demonstrated that conditional knockout (Suryadevara et al. 2024) of *Meis1* in murine CMs extended the postnatal proliferative window and reactivated adult CMs to re-enter mitosis without compromising cardiac function. Conversely, *Meis1* overexpression in neonatal mouse CMs suppressed proliferation by upregulating CDK inhibitors, particularly *Cdkn1a*, thereby impairing neonatal heart regeneration. Notably, *Meis2* silencing post-MI reduced fibrotic scar formation, enhanced CM survival, and improved functional recovery (Alam et al. 2019). Collectively, these findings underscore the role of Meis factors in enforcing cell cycle arrest in postnatal CMs, positioning them as critical targets for modulating cardiac regenerative capacity.

 Hoxb13, a homeodomain transcription factor, functions as a critical regulator of prostate epithelial cell differentiation and cell cycle modulation. In cardiac development and regeneration, Hoxb13 cooperates with Meis1 in postnatal CMs, synergistically governing CM maturation, proliferation, and cell cycle dynamics. Notably, CM-specific *Hoxb13* deletion prolongs postnatal CM proliferative capacity and reactivates cell cycle re-entry in adult hearts. Nguyen et al*.* (2020) demonstrated that dual knockout of *Meis1* and *Hoxb13* in adult mice robustly enhanced CM proliferation, promoted mitotic activity through sarcomere disassembly, and improved left ventricular contractility post-MI, highlighting their cooperative role in cell cycle progression and cardiac regeneration. Mechanistic studies further revealed that the Meis1-Hoxb13 complex binds to promoters of cell cycle regulatory genes (e.g., *p21*, *p27*), recruiting histone deacetylases to maintain chromatin condensation and suppress regeneration-associated transcriptional programs in adult mammalian hearts (Mahmoud et al. 2013; Nguyen et al. 2020). Intriguingly, structural screening of the Meis1/Hoxb13-DNA complex identified baromomycin and neomycin as pharmacological inhibitors that disrupt Meis1-DNA interactions, thereby attenuating transcriptional repression and inducing CM proliferation in vitro. In preclinical models of cardiac ischemia/reperfusion injury, combinatorial administration of these compounds significantly enhanced myocardial regeneration, attenuated fibrosis, and restored cardiac function in both adult mice and pigs (Ahmed et al. 2024). These findings position the Meis1-Hoxb13 axis as a druggable target for therapeutic intervention in cardiac repair.

### GATA4

GATA4, a core transcription factor governing cardiac development and regeneration, exhibits dynamic spatiotemporal activity during myocardial repair. In zebrafish, subepicardial ventricular CMs rapidly upregulate *gata4* within one week post-cardiac injury, forming clonally expanding cell clusters that orchestrate myocardial regeneration at the lesion periphery (Kikuchi et al. 2010). Mechanistically, GATA4 drives cardiac-specific gene activation (e.g., *Myh6, Nppa*) through synergistic interactions with NKX2-5 and TBX5 (Hiroi et al. 2001; Molkentin et al. 1998), while concurrently suppressing Hippo pathway signaling. This inhibition prevents YAP/TAZ phosphorylation, facilitating their nuclear translocation and subsequent upregulation of proliferative markers (CTGF, CYR61) to reinitiate CM cell cycle re-entry (Heallen et al. 2011). Furthermore, GATA4 enhances tissue repair via paracrine regulation, stimulating the secretion of angiogenic factors (FGF16, FGF2, VEGF) to promote neovascularization and optimize the regenerative microenvironment (Bisping et al. 2006; Oka et al. 2007). Although adenovirus-mediated *Gata4* overexpression reduces infarct size in murine MI models, sustained hyperactivation may trigger pathological hypertrophy, underscoring the necessity for precise expression control (Liang et al. 2001; Oka et al. 2007). Additionally, GATA4 activates pro-regenerative pathways such as IL-13 production via NF-κB signaling—a process indispensable for cardiac repair in zebrafish, as NF-κB blockade abolishes regeneration (Karra et al. 2015). Notably, GATA4 serves as a linchpin in cellular reprogramming strategies: its combination with *Mef2c* and *Tbx5* (GMT cocktail) enables direct transdifferentiation of cardiac fibroblasts into functional CMs, offering a transformative approach to mitigate fibrosis and restore cardiac architecture (Ieda et al. 2010).

### TBX20

T-box transcription factor 20 (TBX20), an evolutionarily conserved regulator of cardiac development, is essential for heart morphogenesis and cellular differentiation. Following cardiac injury in zebrafish, *Tbx20* is rapidly upregulated at wound borders, where it orchestrates CM proliferation via dedifferentiation induction (Fang et al. 2020). Concurrently, TBX20 activates endocardial cells in the injured area through non-cell-autonomous mechanisms, enhancing their protrusive activity and proliferation to initiate the BMP6 signaling cascade—a pathway critical for endocardial regeneration, as *Bmp6* inhibition markedly impairs this reparative process (Fang et al. 2020).

In adult mammalian systems, TBX20 demonstrates therapeutic potential. Specific overexpression of *Tbx20* in adult mouse CMs markedly enhances CM proliferation, reduces post-MI infarct size, and improves cardiac function. These effects are mediated through dual mechanisms: activation of pro-proliferative pathways (e.g., AKT/YAP/BMP signaling) and direct suppression of cell cycle inhibitors (*p21*, *Meis1*, *Btg2*), alongside induction of capillary formation and fetal-type contractile protein expression (Xiang et al. 2016). Notably, TBX20 serves as a critical enhancer of cardiac fibroblast reprogramming. Tang et al*.* (2022)demonstrated that supplementing the MGT133 reprogramming cocktail (*MEF2C*, *GATA4*, *TBX5*, miR-133) with TBX20 significantly boosts the conversion efficiency of cardiac fibroblasts into functional CMs. This discovery not only elucidates TBX20’s pivotal role in cellular reprogramming but also advances innovative therapeutic approaches for myocardial regeneration.

### E2F

The E2F transcription factor family (E2F1-E2F8) exhibits multifaceted regulatory functions in cardiac regeneration, orchestrating cell cycle dynamics, metabolic homeostasis, and fibrotic remodeling (Ebelt et al. 2006). Mechanistically, distinct E2F members govern divergent biological processes: E2F1 and E2F3a directly activate cell cycle genes (*Cyclin A2/CDK1*) and synergize with the Hippo-YAP pathway to drive CM cycle re-entry (Wei et al. 2024a). In vivo studies demonstrate that AAV9-mediated *E2f1* overexpression enhances CM proliferation and attenuates fibrosis in murine MI models (Mazurek et al. 2024). Meanwhile, E2F6, a transcriptional repressor, displays developmental stage-specific regulation. Although its mRNA (including splice variant *E2f6b*) persists from embryonic (E18) to adult CMs, protein levels decline postnatally, suggesting posttranscriptional regulation (Movassagh et al. 2006). Knockdown of *E2f6* in neonatal CMs severely compromises cell survival, implicating its role in maintaining physiological CM growth (Movassagh et al. 2006). Concurrently, the E2F pathway regulates myocardial metabolism by suppressing glycolysis through cyclin B1 and prematurely activating the ketolytic enzyme BDH1—an early HF biomarker that modulates connexin-43 via ketone signaling in dilated cardiomyopathy (Major et al. 2017). In addition, a positive feedback loop between *E2f1* and *ECRAR* contrasts with *E2f8*’s inhibitory effects on transcriptional activation, highlighting intra-family functional antagonism that fine-tunes regenerative outcomes (Chen et al. 2019).

### PITX2

Paired-like homeodomain transcription factor 2 (PITX2), a homeodomain-containing transcription factor, serves as a master regulator of left–right asymmetry patterning and organ morphogenesis. Beyond its developmental roles in regulating the differentiation of cardiac neural crest cells and the spatial arrangement of CMs, PITX2 governs mitochondrial metabolism and redox homeostasis during cardiac repair through dual mechanisms: (1) preserving mitochondrial oxidative phosphorylation capacity and (2) activating antioxidant defense systems (Franco and Campione 2003). Clinically, *PITX2* deficiency is implicated in atrial fibrillation predisposition (Kirchhof et al. 2011). In Hippo-deficient murine models, cardiac injury-activated PITX2 cooperates with YAP and NRF2 to drive mitochondrial electron transport chain gene expression and ROS scavenging, essential for CM regeneration. Both *Pitx2* loss and antioxidant system disruption impair cardiac repair, whereas functional restoration rescues regenerative capacity (Tao et al. 2016). CM-specific *Pitx2* knockout (*Pitx2* mice post-MI exhibit ectopic cardiac adipose deposition due to mitochondrial dysfunction. Pitx2 maintains redox equilibrium by regulating mitochondrial targets (e.g., *Cox7c*), thereby inhibiting adipogenic signaling and preserving tissue composition during regeneration (Li et al. 2018a). Recent work by Chen et al*.* reveals that cardiac NRF3 exacerbates CM apoptosis by suppressing *Pitx2* expression, elevating mitochondrial ROS. Targeting the NRF3-PITX2-ROS axis emerges as a novel therapeutic strategy for MI (Chen et al. 2025a). These findings provide an important theoretical basis for the development of therapeutic strategies for cardiac regeneration based on Pitx2 regulation.

### Other transcription factors

During cardiac regeneration, multiple other TFs are involved in a sophisticated network through spatiotemporal-specific regulatory mechanisms. Forkhead Box M1 (FOXM1) emerges as a central regulator, directly activating cell cycle genes (e.g., *Ccnb1*, *Cdk1*) to propel CMs into proliferative phases. Cooperative activation of its downstream target centrosomal microtubule-binding protein (Cenpf) ensures precise chromosomal segregation and cytokinesis, collectively establishing the Foxm1-Cenpf axis as indispensable for regenerative fidelity (Zuppo et al. 2023). Parallel studies in zebrafish reveal Klf1’s unique capacity to initiate CM dedifferentiation via DNA methylation remodeling, particularly through demethylation of *gata4* and *tbx5* enhancer regions, thereby restoring proliferative potential (Ogawa et al. 2021). Notably, this epigenetic reprogramming paradigm finds functional convergence in mammals: targeted activation of the OCT4/SOX2/KLF4/C-MYC network circumvents terminal differentiation barriers in adult CMs, inducing coordinated epigenetic resetting and metabolic rewiring to achieve functional regeneration (Chen et al. 2021).

The regulatory landscape further integrates stress-responsive checkpoints. TRP53 constrains CM proliferation and hypertrophic growth, while its negative regulator MDM2 counteracts this suppression. Mitogenic factors NRG1/Vegfaa amplify proliferative signals through strategic activation of the Mdm2-Tp53 axis (Shoffner et al. 2020). Complementing this balance, NRF1 orchestrates dual protective mechanisms in regenerating CMs—activating proteasomal function and redox homeostasis to drive regeneration-associated transcription. Genetic ablation of *Nrf1* disrupts this transcriptional program, whereas its overexpression confers protection against ischemia/reperfusion injury by sustaining stress adaptation (Cui et al. 2021). Single-cell sequencing advances have identified regeneration-associated CM4 subpopulations governed by nuclear transcription factor Y subunit alpha (NRFα) and nuclear factor erythroid 2-like 1 (NFE2L1). Functional validation demonstrates *Nfyα* overexpression enhances CM proliferation, while *Nfe2L1* coactivation synergistically overcomes post-injury regenerative constraints through viability potentiation (Cui et al. 2020).

Metabolic-transcriptional crosstalk further modulates regenerative outcomes. FOXO3 impedes CM proliferation via SFRP2-mediated suppression of Wnt/β-catenin signaling. *FoxO3* deletion accelerates myocardial repair by relieving β-catenin inhibition—an effect reversible by *Sfrp2* overexpression (Xia et al. 2025). Conversely, the Foxp1/USP20/HIF1α/Hand1 axis negatively regulates proliferation through metabolic gatekeeping: injury-induced *Foxp1* downregulation promotes regeneration by shifting energy metabolism from fatty acid oxidation to glycolytic flux. Therapeutic delivery of AAV9-cTnT-Hand1 activates this pathway, significantly enhancing functional recovery (Wang et al. 2025).

Collectively, these transcriptional regulators exhibit dual ontogenetic significance—not only orchestrating embryonic cardiogenesis but also harboring transformative potential for adult cardiac repair. Their coordinated actions illuminate conserved molecular bridges linking developmental patterning with regenerative plasticity, offering multifaceted therapeutic entry points for heart regeneration.

**Fig. 4:**
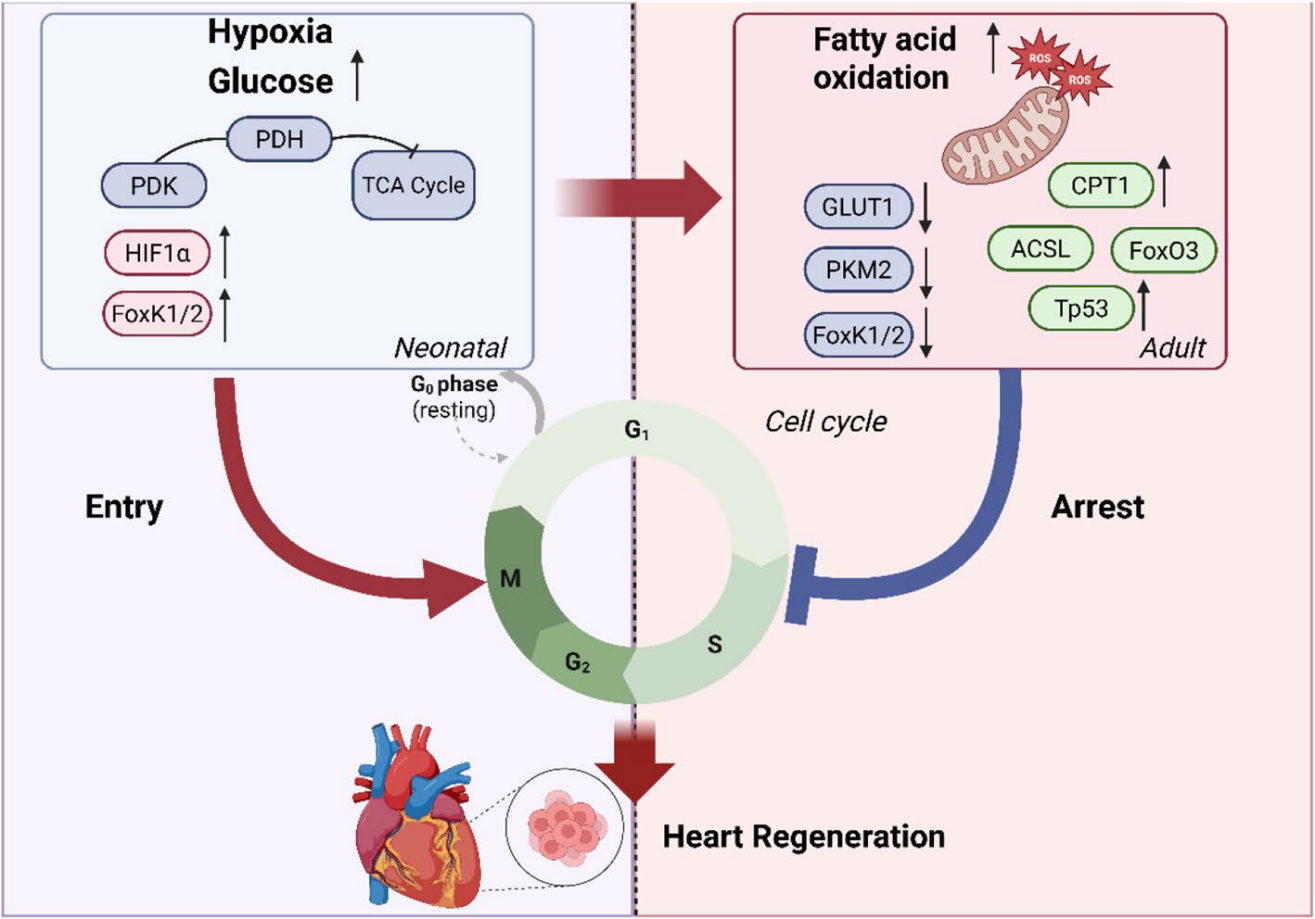
Integration of metabolic states with cell cycle entry in CMs. In neonatal CMs, hypoxia and high glucose availability upregulate HIF-1α and FoxK1/2, driving a glycolytic metabolic state that promotes cell cycle entry and heart regeneration. Conversely, adult CMs rely on FAO driven by CPT1, ACSL, FoxO3, and TP53, which sustains cell cycle arrest and limits regenerative capacity.Created in https://BioRender.com

The relationship between metabolism, cell cycle, and cardiac regeneration is summarized in the following figure (Fig. 4).

## Cell cycle proteins

Adult mammalian CMs gradually exit the cell cycle after birth and exhibit polyploid or multinucleated features (Alkass et al. 2015; Li et al. 1996; Soonpaa et al. 1996). And polyploid and multinucleated myocytes lose their ability to proliferate and regenerate (Kirillova et al. 2021). The expression of cell cycle activators (e.g., cyclins A, B1, D1, and E; and CDK1/2/4) was significantly down-regulated from embryonic to postnatal stages, whereas inhibitors p21, p27, and RB were consistently up-regulated (Ahuja et al. 2007; Kang and Koh 1997; Koh et al. 1998; Poolman et al. 1998). In the mouse model, the expression of cell cycle proteins and CDK progressively decreased from embryonic E12/E16 to birth (P0), followed by a transient rebound at P5 (Ikenishi et al. 2012). Enhanced expression of the CDK inhibitors p21/p27 with age in the mammalian heart further consolidates the state of cycle arrest, whereas pocket protein RB directly drives CM terminal differentiation by inhibiting E2F transcriptional activity (Bergmann et al. 2009). Cell cycle protein D2 (*Ccnd2*) overexpression significantly promoted DNA synthesis and infarct repair in adult mouse CMs, whereas D1/D3 isoforms had weak effects (Chaudhry et al. 2004; Cheng et al. 2007). Cell cycle protein A2 elevates ejection fraction and induces cytoplasmic division by adenoviral delivery of *Ccna2* in a porcine model of MI (Shapiro et al. 2014). However, single-factor interventions are often accompanied by limitations; for example, although cell cycle protein D1 induces 40% of CMs to re-enter the cycle, most of them stagnate in the M-phase or undergo endoreplication, highlighting the need for precise regulation (Fig. 4).

In response to deficiencies against single-factor therapies, combinatorial gene strategies show higher potential. Co-overexpression of CDK1-CDK4-CCNB-CCND (4F) developed by Mohamed’s team resulted in a 15%−20% increase in proliferation rates of mouse and human CMs, and significant improvement in cardiac function in infarction models (Mohamed et al. 2018; Tane et al. 2014). In recent years, Jianyi Zhang’s team has innovatively developed CM-SMRTs to achieve transient and specific overexpression of *Ccnd2* in small and large mammalian acute MI models through CCND2-SMRTs. This technology induced a significant increase in CM proliferation in the infarct zone and improved left ventricular function without long-term safety risks, providing an efficient and controlled delivery platform for clinical translation (Sun et al. 2023). These advances suggest new pathways for cardiac injury repair through multi-targeted synergistic regulation (e.g., activation of cell cycle proteins in conjunction with inhibition of CDK inhibitors) and delivery technologies.

### Non-coding RNAs

**Fig. 5:**
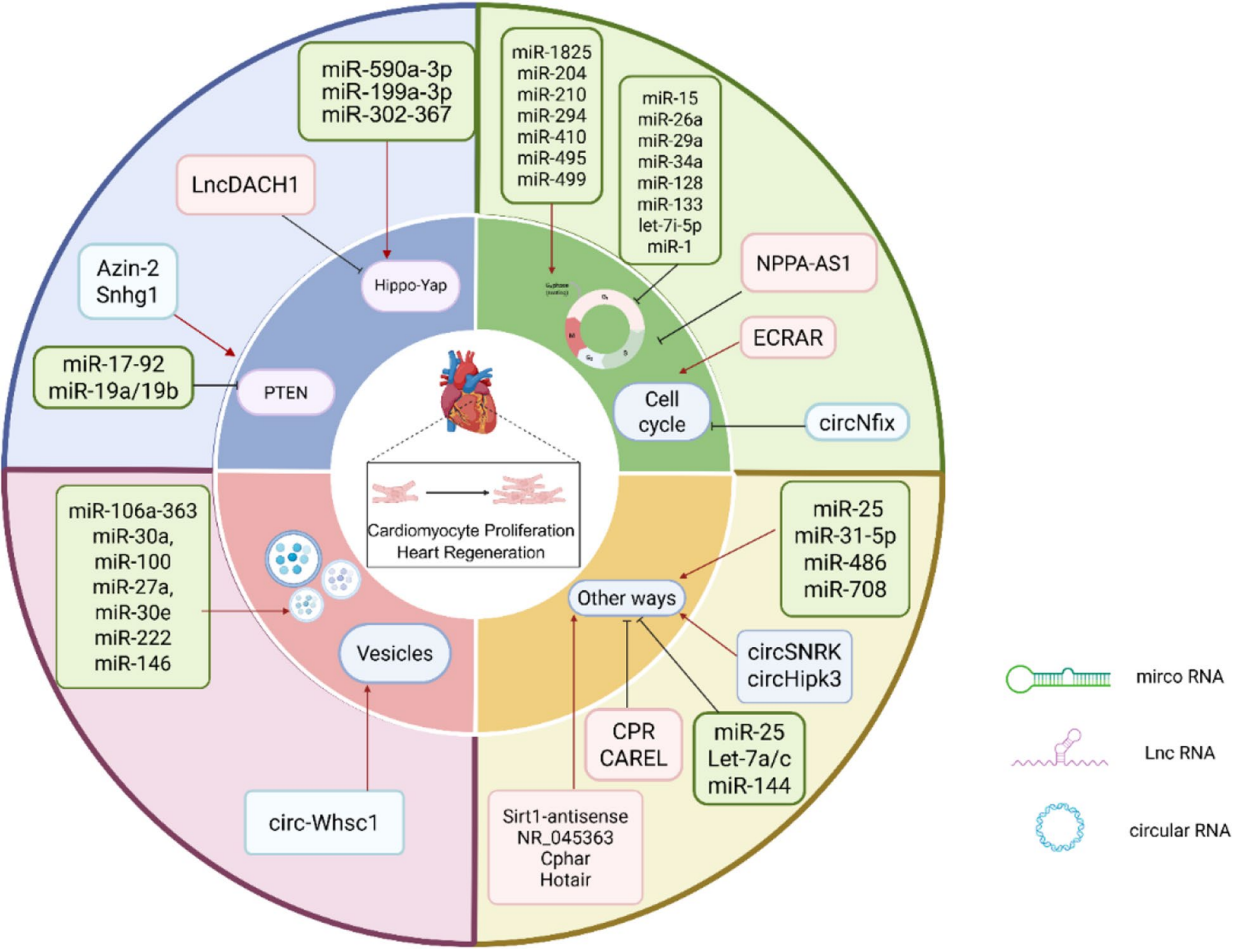
Molecular mechanisms of miRNAs, lncRNAs, and circRNAs in regulating CM proliferation and promoting cardiac regeneration following injury. Created in https://BioRender.com

Recently, a growing body of research has demonstrated the involvement of non-coding RNAs (ncRNAs)—including microRNAs (miRNAs), long non-coding RNAs (lncRNAs), and circular RNAs (circRNAs)—in the regulation of CM proliferation and regeneration (Fig. 5).

Notably, many miRNAs that promote CM proliferation converge on the Hippo signaling pathway. For instance, miR-302/367 downregulates MST1, LATS2, and MOB1, while miR-199a-3p suppresses TAOK1 and BTRC, thereby activating YAP to promote CM proliferation (Diez-Cuñado et al. 2018). Beyond the Hippo pathway, several pro-proliferative miRNAs promote CM proliferation by downregulating key inhibitors, including the CDK inhibitor p21 (e.g., the miR-302/209 family) and the tumor suppressor PTEN (e.g., the miR-17-92 cluster, miR-486, and miR-19a/19b) (Gao et al. 2019); miR-25 enhances proliferation in neonatal rat CMs by inhibiting the small GTPase RhoBTB1 (Qin et al. 2019); and miR-486 stimulates CM proliferation by indirectly reducing FoxO1 and Smad signaling while increasing STAT1 expression associated with Gata4 and serum response factor (Lange et al. 2019).

Similarly to miRNAs, lncRNAs have been shown to regulate CM proliferation either by enhancing or suppressing cell cycle progression. LncRNAs often function as competitive endogenous RNAs that sequester miRNAs and modulate their activity. For example, the lncRNA AZIN2-SV, a splice variant of the AZIN2 gene, directly binds to miR-214 and blocks its inhibitory effect on *Pten*, so loss of AZIN2-SV promotes CM proliferation and survival (Li et al. 2018b). Some other lncRNAs, such as CAREL, CRRL, CPR, and DACH1, also suppress CM proliferation (Yuan and Krishnan 2021). Similarly, lncRNA NR-045363 acts as a sponge for miR-216a, thereby promoting CM proliferation via the JAK/STAT3 pathway. Conversely, suppression of LUCAT1 inhibits CM proliferation and induces apoptosis through the miR-612/HOXA13 axis (Wang et al. 2019a). Recently, more lncRNAs have been reported to promote CM proliferation, such as ECRAR, Sirt1 AS lncRNA (Yuan and Krishnan 2021). In addition, other lncRNAs (such as Meg3, H19, LncRNA-ROR) also regulate CM apoptosis, differentiation, or reprogramming to contribute to cardiac regeneration.

circRNAs, characterized by their covalently closed loop structure formed via back-splicing, exhibit greater stability than linear RNAs and play significant regulatory roles in cardiac regeneration (Qu et al. 2015). For example, circSNRK absorbs miR-103-3p to upregulate SNRK, which subsequently binds to GSK3β and promotes cardiac regeneration after MI (Zhu et al. 2021). circHipk3 enhances the stability of the NICD through acetylation, thereby stimulating CM proliferation, and also functions as a sponge for miR-133a to increase CTGF expression, ultimately activating endothelial cells and promoting angiogenesis (Si et al. 2020). Similarly, circ-Amotl1 improves CM survival under oxidative stress in vitro, while circFndc3b treatment reduces apoptosis, enhances angiogenesis, and improves left ventricular function post-MI in vivo (Zeng et al. 2017). Currently, circNfix has been identified as an anti-regenerative circRNA that suppresses both CM proliferation and angiogenesis. It interacts with Ybx1 and Nedd4l, leading to Ybx1 ubiquitination and degradation, and also sponges miR-214 to target GSK3β, thereby inhibiting β-catenin signaling and angiogenesis (Huang et al. 2019). In contrast, circHipk3, which is highly expressed in fetal and neonatal hearts, promotes both CM proliferation and angiogenesis (Si et al. 2020).

However, the role of circRNAs in cell cycle regulation remains relatively limited. At this stage, ncRNAs are increasingly important in regulating CM proliferation and cardiac regeneration after injury in cardiac diseases. ncRNAs regulate myocardial proliferation by targeting key proteins as well as epigenetic modifications. The combination of precision technologies such as spectral tracing, novel delivery approaches such as exosomes/synthetic hydrogels, modified mRNAs, and large libraries of uncharacterized RNA functions opens up a new dimension for the development of RNA therapeutics. Although the therapeutic potential of miRNAs is becoming clear, the same potential of lncRNAs and circRNAs for cardiac regeneration in humans remains to be determined (Wang et al. 2021b). This paper focuses on the overall mechanistic framework of signaling pathways and the specific molecular details of ncRNAs and related therapeutic modalities can be found in other excellent literature (Abbas et al. 2020; Braga et al. 2021).

### Methodological advances in cardiac regeneration research

The convergence of single-cell transcriptomics, multi-omics profiling, and computational structural biology is fundamentally transforming our ability to discover and validate therapeutic targets for cardiac regeneration.

Bulk RNA-seq quantifies gene expression across tissues, enabling unbiased discovery of differentially expressed genes. In cardiac regeneration, comparing proliferative and non-proliferative states has identified critical regulators. Comparative RNA-seq between cycling and non-cycling CMs uncovered LRP6 as a proliferation brake acting through the ING5/p21 axis. Transcriptome profiling of neonatal mouse hearts after resection also revealed OSM as a top-ranked upstream regulator of CM proliferation. (Li et al. 2020b; Wu et al. 2021)

Multi-omics integration combines complementary layers—such as transcriptomics with ATAC-seq or Ribo-seq—to capture mechanisms invisible to transcriptomics alone. Using RNA-seq coupled with ATAC-seq, *KLF1* was shown to promote heart repair by simultaneously activating cell-cycle genes and repressing fatty acid metabolism at both transcriptional and epigenetic levels. Employing Ribo-seq alongside RNA-seq, *NAT10* was found to enhance cardiac regeneration through translational control of oxidative phosphorylation genes. (Hao et al. 2025; Ma et al. 2024)

Single-cell and spatial transcriptomics resolve expression at individual cell resolution, overcoming the averaging of bulk methods to reveal cellular heterogeneity. High-resolution scRNA-seq integrated with spatial transcriptomics (Stereo-seq) generated a zebrafish heart regeneration atlas, identifying *angpt4* as a conserved regulator initiating an epicardial-endothelial-CM coordination cascade. Single-nucleus RNA-seq of YAP-activated hearts further demonstrated that brief YAP stimulation reshapes the regenerative microenvironment by enriching repair-associated fibroblasts and anti-inflammatory macrophages. (Liu et al. 2026; Wu et al. 2023)

High-content screening and computational structural biology address how to pharmacologically target regulators identified by omics. High-content screening combines automated imaging with computer vision to evaluate compound libraries against cellular phenotypes. An EdU-based screen identified Nimodipine as an L-type calcium channel inhibitor that promotes CM proliferation through LRP5-dependent Wnt activation. Molecular dynamics simulations guided the discovery that Salvianolic acid B binds PHB1, inducing conformational changes that enhance PHB1-Raf interaction and activate Raf-ERK signaling to promote CM mitosis (Cao et al. 2020; Feng et al. 2026).

Collectively, these advanced methodologies have transformed target discovery from gene-by-gene hunting to systematic, data-driven prioritization.

### Conclusions

Cardiac regeneration represents a multifaceted biological process, including CM proliferation, cardiac fibrosis, neovascularization, immune response and energy metabolism (Weinberger and Riley 2024), which requires coordinated interactions among diverse cell types and signaling cascades. A prominent therapeutic strategy for HF involves harnessing the endogenous proliferative capacity of CMs, as well as other related processes of cardiac regeneration. Therefore, the discovery of the molecular and cellular mechanisms of CM proliferation and cardiac regeneration is a key for the development of potential therapy for heart injury, such as MI.

Nowadays, emerging technologies are advancing regenerative medicine paradigms. For instance, engineered myocardial grafts combining iPSC-derived CMs and stromal cells, implanted in HF-afflicted non-human primates under immunosuppression, demonstrated 6-month retention and dose-dependent functional recovery via myocardial regeneration (Jebran et al. 2025). Mechanical innovations, such as left ventricular assist devices, reduce cardiac load and have been shown to enhance CM renewal rates by sixfold, providing a promising foundation for cell-free combinatorial therapies (Derks et al. 2023). Additionally, inhaled stem cell exosome nebulization therapy improved cardiac function in preclinical models by reprogramming glucose metabolism via CD36 downregulation (Li et al., 2024). Cardiac in vivo reprogramming, which has been achieved using TFs, miRNAs, and/or small molecules, also provides a potential opportunity for cardiac regeneration (Xie et al. 2023). Beyond engineered grafts and modified mRNA, emerging human cardiac organoid and microfluidic “organ-on-a-chip” platforms offer complementary advantages in cardiac regeneration research, enabling the recapitulation of myocardial injury and repair processes in a human-relevant 3D microenvironment (Arslan et al. 2024). For instance, integrating melt-electrowriting with microfluidics has been shown to engineer a human cardiac microenvironment that supports high-fidelity drug screening and regeneration-oriented compound evaluation (Jones et al. 2025). Furthermore, electroconductive heart-on-a-chip models have demonstrated the ability to enhance CM maturation and contractile function, providing a powerful platform to bridge the translational gap between animal models and clinical applications (Esmaeili et al. 2025). However, many challenges remain.

Additionally, despite progress, several critical knowledge gaps persist. First, the spatiotemporal thresholds for achieving clinically meaningful CM proliferation and regeneration in adult mammalian hearts remain undefined. Second, challenges in therapeutic delivery persist, as current gene therapies risk off-target effects and immunogenicity. Third, translational limitations arise from pathophysiological disparities between experimental murine models and human cardiac disease. Fourth, the mechanistic links between metabolic reprogramming, epigenetic modifications, and regenerative outcomes require further elucidation. The future of cardiac regeneration will no longer be limited to a single technological breakthrough. By integrating multi-omics insights, smart biomaterials and interdisciplinary approaches to bridge the gap between transient repair and functional cardiac reconstruction.

Among the emerging translational strategies, hiPSC-CM-based transplantation and Hippo-targeted gene therapy (e.g., AAV9–Sav–shRNA) have entered or are approaching clinical testing, supported by large animal models (pig/sheep). However, several translational barriers remain. First, long-term engraftment stability, specifically functional integration and host–graft interface maturation, is insufficiently characterized. Second, the scalability of cell-based therapies, including GMP-compliant production, cryopreservation, and off-the-shelf availability, poses a major hurdle. Third, complications such as arrhythmogenicity and exacerbated fibrosis require systematic long-term safety monitoring in large animals. Fourth, heterogeneity in preclinical outcomes across studies underscores the need for standardized validation pipelines. Collectively, these efforts are building a bridge between preclinical innovation and clinical translation, advancing toward therapies that address the unmet need for cardiac regeneration in heart failure.

## Data Availability

Not applicable.

## Abbreviations

ACE: Angiotensin-converting enzyme
ACTRT2: Actin-related protein T2
ADAM10: A disintegrin and metalloproteinase domain-containing protein 10
AKT: Protein kinase B
AMPK: AMP-activated protein kinase
Ang-1: Angiopoietin-1
ANP: Atrial natriuretic peptide
APC: Adenomatous polyposis coli
ATAC-seq: Assay for transposase-accessible chromatin using sequencing
ATF4: Activating transcription factor 4
AAV9: Adeno-associated virus serotype 9
Axin: Axis inhibition protein
Bax: BCL2-associated X protein
BDH1: 3-Hydroxybutyrate dehydrogenase 1
BMP: Bone morphogenetic protein
BMPR: Bone morphogenetic protein receptor
BTRC: Beta-transducin repeat containing E3 ubiquitin protein ligase
CBP: CREB-binding protein
CCNB1: Cyclin B1
CCND2: Cyclin D2
CDK: Cyclin-dependent kinase
CDKN1A: Cyclin-dependent kinase inhibitor 1A
cDNA: Complementary DNA
ceRNA: Competing endogenous RNA
Cenpf: Centrosomal microtubule-binding protein
CHK1: Checkpoint kinase 1
circRNA: Circular RNA
CK1: Casein kinase 1
c-Met: Mesenchymal-epithelial transition factor receptor
c-Myc: MYC proto-oncogene
CM: Cardiomyocyte
CM-SMRTs: Cardiomyocyte-specific modified mRNA translation system
CPT1b: Carnitine palmitoyl transferase 1b
CREB1: cAMP responsive element-binding protein 1
CRISPR-Cas9: Clustered regularly interspaced short palindromic repeats-associated protein 9
CRLR: Calcitonin receptor-like receptor
CSL: CBF1/Su(H)/Lag-1
CTGF: Connective tissue growth factor
CYR61: Cysteine-rich angiogenic inducer 61
DACH1: Dachshund family transcription factor 1
DAG1: Alpha-dystroglycan
DDR: DNA damage response
DKK1: Dickkopf WNT signaling pathway inhibitor 1
DLL: Delta-like ligand
DNMT1: DNA methyltransferase 1
DUSP: Dual-specificity phosphatase
ECM: Extracellular matrix
EGF: Epidermal growth factor
ERK: Extracellular signal-regulated kinase
FAO: Fatty acid oxidation
FGF: Fibroblast growth factor
FGFR: Fibroblast growth factor receptor
FMN2: Formin 2
FOXK1: Forkhead box K1
FOXK2: Forkhead box K2
FOXO: Forkhead box O
FOXM1: Forkhead box M1
FOXP1: Forkhead box P1
FRMD: FERM domain-containing protein
FSTL1: Follistatin-like 1
GATA4: GATA binding protein 4
GLUT1: Glucose transporter 1
GMP: Good manufacturing practice
GMT: GATA4, MEF2C, TBX5
gp130: Glycoprotein 130
GR: Glucocorticoid receptor
GSK-3β: Glycogen synthase kinase-3 beta
HAND1: Heart and neural crest derivatives expressed 1
HF: Heart failure
HGF: Hepatocyte growth factor
HIF: Hypoxia-inducible factor
hiPSC-CM: Human induced pluripotent stem cell-derived cardiomyocyte
HMGCS2: 3-Hydroxy-3-methylglutaryl-CoA synthase 2
HOXB13: Homeobox B13
hCVPCs: Human cardiovascular progenitor cells
hCMs: Human cardiomyocytes
HSPG: Heparan sulfate proteoglycan
IGF: Insulin-like growth factor
IGF2BP3: Insulin-like growth factor 2 mRNA-binding protein 3
IL-13: Interleukin-13
IND: Investigational new drug
iPSC: Induced pluripotent stem cell
JAG: Jagged ligand
JAK: Janus kinase
JNK1: c-Jun N-terminal kinase 1
KLF: Kruppel-like factor
LATS: Large tumor suppressor
LDHA: Lactate dehydrogenase A
LEF: Lymphoid enhancer factor
lncRNA: Long non-coding RNA
LRP: Low-density lipoprotein receptor-related protein
LUCAT: Lung cancer associated transcript
MAPK: Mitogen-activated protein kinase
MEF2C: Myocyte enhancer factor 2C
MEIS1: Meis homeobox 1
MEK: Mitogen-activated protein kinase kinase
MHC: Myosin heavy chain
MI: Myocardial infarction
miRNA: MicroRNA
MMP: Matrix metalloproteinase
MOB1: MOB kinase activator 1
mRNA: Messenger RNA
MST: Mammalian Ste20-like kinase
mTOR: Mammalian target of rapamycin
MYDGF: Myeloid-derived growth factor
NADH: Nicotinamide adenine dinucleotide (reduced)
NEDD4L: Neural precursor cell expressed developmentally down-regulated 4-like
NF-κB: Nuclear factor kappa-light-chain-enhancer of activated B cells
NFE2L1: Nuclear factor erythroid 2-like 1
NFYA: Nuclear transcription factor Y subunit alpha
NGF: Nerve growth factor
NICD: Notch intracellular domain
NKX2-5: NK2 homeobox 5
NOGO-B: Neurite outgrowth inhibitor-B
NRF1: Nuclear respiratory factor 1
NRG: Neuregulin
OCT4: Octamer-binding transcription factor 4
OSM: Oncostatin M
OSMR: Oncostatin M receptor
p38: p38 mitogen-activated protein kinase
p70S6K: p70 S6 kinase
PARP1: Poly(ADP-ribose) polymerase 1
PDGF: Platelet-derived growth factor
PDGFR: Platelet-derived growth factor receptor
PDK: Pyruvate dehydrogenase kinase
PHB1: Prohibitin 1
PHLDA1: Pleckstrin homology like domain family A member 1
PI3K: Phosphoinositide 3-kinase
PIM1: Pim-1 proto-oncogene
PITX2: Paired-like homeodomain transcription factor 2
PKM2: Pyruvate kinase M2
PKP4: Plakophilin 4
PP1: Protein phosphatase 1
PP2A: Protein phosphatase 2A
PPAR: Peroxisome proliferator-activated receptor
PTEN: Phosphatase and tensin homolog
pRASSF1A: Ras association domain family member 1A
Ribo-seq: Ribosome profiling sequencing
ROS: Reactive oxygen species
S1P: Sphingosine-1-phosphate
SAV1: Salvador family WW domain-containing protein 1
ppscRNA-seq: Single-cell RNA sequencing
SERCA: Sarcoplasmic/endoplasmic reticulum calcium ATPase
SFRP2: Secreted frizzled-related protein 2
SMAD: Mothers against decapentaplegic homolog
SMRTs: Modified mRNA translation system
SphK: Sphingosine kinase
SRF: Serum response factor
ssTnI: Skeletal troponin I
STAT: Signal transducer and activator of transcription
TAOK1: Thousand and one amino acid kinase 1
TAZ: Transcriptional coactivator with PDZ-binding motif
TBX: T-box transcription factor
TCF: T-cell factor
TEAD: TEA domain transcription factor
TF: Transcription factor
TLR: Toll-like receptor
TNF-α: Tumor necrosis factor alpha
TWEAK: Tumor necrosis factor-like weak inducer of apoptosis
USP20: Ubiquitin specific peptidase 20
VEGF: Vascular endothelial growth factor
VEGFR: Vascular endothelial growth factor receptor
WBP2: WW domain-binding protein 2
Wnt: Wingless-related integration site
YAP: Yes-associated protein
YBX1: Y-box binding protein 1/p
